# Harnessing the complexity of gene expression data from cancer: from single gene to structural pathway methods

**DOI:** 10.1186/1745-6150-7-44

**Published:** 2012-12-10

**Authors:** Frank Emmert-Streib, Shailesh Tripathi, Ricardo de Matos Simoes

**Affiliations:** 1 Computational Biology and Machine Learning Laboratory, Queen’s University Belfast, Belfast, UK

**Keywords:** Gene expression data, Cancer data, Statistical analysis methods, Pathway methods, Correlation structure, Cancer genomics

## Abstract

High-dimensional gene expression data provide a rich source of information because they capture the expression level of genes in dynamic states that reflect the biological functioning of a cell. For this reason, such data are suitable to reveal systems related properties inside a cell, e.g., in order to elucidate molecular mechanisms of complex diseases like breast or prostate cancer. However, this is not only strongly dependent on the sample size and the correlation structure of a data set, but also on the statistical hypotheses tested. Many different approaches have been developed over the years to analyze gene expression data to (I) identify changes in single genes, (II) identify changes in gene sets or pathways, and (III) identify changes in the correlation structure in pathways. In this paper, we review statistical methods for all three types of approaches, including subtypes, in the context of cancer data and provide links to software implementations and tools and address also the general problem of multiple hypotheses testing. Further, we provide recommendations for the selection of such analysis methods.

**Reviewers:**

This article was reviewed by Arcady Mushegian, Byung-Soo Kim and Joel Bader.

## Review

### Background

The early driving forces in biology were reductionist approaches. In general, a reductionist approach tries to break-down a complex system into its parts list and explains its properties as the sum of its individual components. Hence, the individual constituents of a system inform its higher level functions [1-4]. However, the ‘one gene, one protein, one function’ working hypothesis [5] is not sufficient in order to explain the many emergent properties such as the phenotypic variability of organisms or the heterogeneity of cancer [6]. For this reason, nowadays, it is generally acknowledged that for achieving a functional understanding of biological systems, the genes in a cell need to be studied as a functioning collective [2,3,7]. In such a system, the collective functioning of groups of genes results in, for instance, signaling pathways or protein complexes that regulate cell differentiation, transcription regulation or growth.

A systems integration at the cellular level has the potential to answer many, until now, unsolved questions about biological systems and their collective functioning, regulatory programs for growth, development, phenotypic variability and the causality of many complex diseases [8-10]. Due to the enormous complexity of a cellular system, where many processes and interactions at different levels inside a cell work in harmony to assure the vital functioning of a cell, we need to understand key properties of biological systems like its robustness or modularity [2,8] in order to enhance our understanding of complex diseases. These complex interactions occurring within a cell can be described by networks [11-13], including gene regulatory networks [14,15], protein-protein interaction (PPI) networks [16,17], metabolic networks [18] and transcription regulatory networks [19,20]. The networks are organized at different cellular levels and enable the functionality of the cell. The question now arising is how can the complexity inside a cell be understood, and analyzed?

The development of information processing technologies in the post genomic era enabled the generation of huge amounts of data. In this review, we focus on gene expression data from microarray platforms and summarize three major types of analysis strategies: (I) Identification of changes in single genes, (II) identification of changes in gene sets or pathways, and (III) identification of changes in the correlation structure within pathways. We discuss these methods in the context of cancer data sets to emphasize their biological meaning, implications and expressiveness.

## Large-scale gene expression data

In the next section, we briefly review high-throughput technologies that enable the generation of large-scale gene expression data [21-23].

### Gene expression data from microarray

A microarray experiment measures genome-wide gene expression levels of mRNA in a cell or a tissue sample under a particular condition. A microarray chip quantifies the hybridization of fluorecsent labeled target nucleotide sequences to defined complementary probe sequences that are spotted on a glass or silicon slide. For different microarray platforms the spotted probes are synthetic oligonucleotides ranging from 25 to 80 nucleotides or long cDNA transcripts. Different microarray platforms were designed for a single-channel or a multi-channel experimental setting. For single-channel arrays each condition sample is hybridized separately on individual arrays using a single dye. For multi-channel arrays multiple conditions are hybridized together on individual arrays using multiple dyes. For example Affymetrix is a single-channel platform, where multiple oligonucleotide probes (probeset) of 25 bases are used to measure the concentration of a mRNA transcript. The target mRNAs of expressed genes are extracted from a treatment or a control sample, reverse transcribed to cDNAs, labeled with a fluorescent dye and then hybridized to a microarray. An image of the microarray captures laser induced emitted fluoresent intensities of the probes at each spot. The intensities give a proportional measure of the corresponding mRNA concentration for each gene that was defined on the microarray.

### Gene expression data from next generation sequencing (RNA-seq)

The transcriptome of a cell comprises mRNA, tRNA, rRNA, and short regulatory RNAs. RNA-seq is a transcriptome sequencing approach that uses deep sequencing techniques such as 454 (Roche), genome analyzer (Illumina solexa), SOLiD (support oligonucleotide ligation detection), Polonator G.007, HeliScope (Helicos BioSciences) and SMRT (single molecule real time sequencing) [24].

RNA-seq has a wide variety of applications such as the measurement of gene expression levels from transcribed mRNA sequences [25]. In the first step of the procedure RNA is extracted from a given condition sample, fragmented, reverse transcribed to cDNA that is ligated to adapters. In the second step a library of reads is generated from the ligated fragments that are sequenced. In the third step the reads are mapped to known exon sequences of genes. The expression level of a gene is measured from the normalized number of mapped sequences that mapped to the known set of exon sequences of a gene. The RNA-seq transcriptome sequencing approach overcomes several limitations of microarrays for measuring gene expression. For example, RNA-seq measures large ranges of expression levels from very low to highly expressed genes and is able to consider unknown transcribed sequences. Since the novelty of the methodology, gold standard procedures for the management and processing of the data are currently being established.

## Gene expression data and cancer

**Figure 1:**
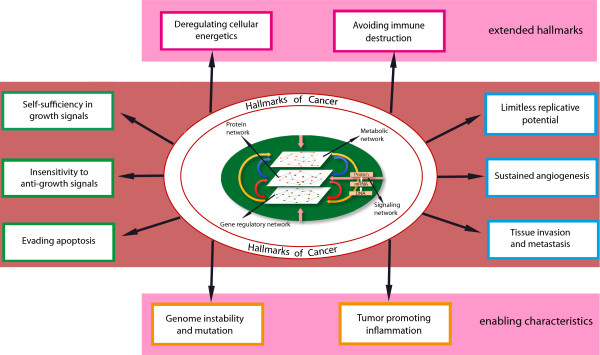
**The hallmarks of cancer and enabling characteristics **[6]**,**[26]**.**

Cancer is a multifactorial disease, i.e., the detection of one mutation in one gene cannot explain the phenotypic plurality of carcino- and pathogenesis by a one-to-one relationship between genotype and phenotype. Instead, cancer can be induced by a multitude of genetic and environmental factors and the accumulation of such events. The intervening of such complex factors makes in general the characterization of complex diseases difficult. For this reason it is astonishing that the seminal work by Weinberg et al. [6,26] presented a relative simple, systematic functional framework for cancer and the role different biological key processes are playing. In this paper, the so called *hallmarks of cancer* have been defined. According to [6], the hallmarks of cancer (see Figure 1) are:

– self sufficiency in growth signals

– insensitivity to anti-growth signals

– evading apoptosis

– limitless replicative potential

– sustained angiogenesis

– tissue invasion and metastasis

Later, this list has been extended by adding two further hallmarks [26]

– deregulating cellular energetics

– avoiding immune destruction

and also two enabling characteristics

– genome instability

– mutation and tumor-promoting inflammation

It has been recognized that these hallmarks are gradually acquired by different types of cancers, potentially, in a variable order. This variability in the acquiring of these disease-bearing processes is one of the indicators of the complexity of cancer.

The biological processes in a cell are controlled and regulated by signaling pathways that are activated by internal and external signaling receptors and factors. The signaling pathways governing growth and cell proliferation are likely dysregulated in their functioning in cancer. For example, they become insensitive to anti-growth signals, or they are dys-regulated in growth signaling pathways by gaining autonomy in their growth. It is assumed that interaction changes at various levels (genetic, mRNA or protein) lead to the unlimited growth of cells instead of the up-regulation or down-regulation of a single gene. Further, sometimes, even a moderate change in the expression of a group of genes can lead to a significant change in the biological function of an organism [27].

Currently, the underlying processes that contribute to cancer are being intensively investigated. However, so far, the molecular causes that initiate and maintain cancer are not well understood. For this reason, the understanding of gene expression profiles, which provide signatures of all the active genes and their interconnections in a cell, contain valuable information about the functioning of key pathways, as expressed by the hallmarks of cancer and, hence, enable a practical investigation of functional mechanisms thereof [28-36]. Despite the different focus of many studies of different cancer types, common themes in the form of ‘key pathways’ can be found throughout. For instance, the NF-*κ*B pathway involved in the cellular responses to external stimuli like cytokines or free radicals, and immune response to infection [29,37-39]; the MAPK signaling pathway responsible for regulating growth factor signaling including the RAF, MEK, and MAPK cascade [34,39,40]; the p53 signaling pathways involved in DNA damage control, apoptosis and inhibition of angiogenesis [37,41,42]; or the Wnt signaling pathway involved in cell differentiation, and cell polarity [31,34,36,38].

## Formulating biological hypotheses

A main goal of high-throughput gene expression analysis is to identify differentially expressed genes or gene sets between two or more conditions to enable a functional interpretation of the underlying condition-specific mechanisms. The biological processes at the gene level are complex in nature as they dynamically interact with each other. A single gene can participate in different biological processes and regulate different genes at different time points. The identification of key genes or pathways is a difficult task, because their interactions are unknown. We only observe the phenotypic outcome of test conditions and the corresponding gene expression patterns measured from a tissue or cell culture. Univariate and multivariate statistical methods can be applied in order to understand such differences from a statistical perspective. The first type of approach that has been used to identify changes in the gene expression is a differential gene expression analysis. This approach is commonly used to compare different conditions of microarray samples to identify differences between them. As a result, a single gene analysis approach gives a list of genes that show a statistically significant difference between two conditions. For cancer, such genes may correspond to oncogenes or tumor suppressor genes.

If we consider the underlying network where different biological functions are being described by groups of interacting genes, a single gene analysis does not resolve the biological functions that are affected primarily in disease conditions and are causal factors of the disease. In order to get a systematic understanding of the disease or phenotypes we have to first understand what biological functions contribute to these changes, and perform a comparison between conditions using *groups of genes* defined by biological pathways. This approach leads to comparing gene expression data at the pathway level where sets of genes are tested for differential expression.

Another interesting property that can be extracted from gene expression data is the correlation structure of gene expression profiles between all genes. This correlation structure shows associations between genes which directly or indirectly interact with each other [8,43-45]. Comparative analyses of gene pathways that consider the correlation structure of expression data can provide a suitable test for the hypothesis of changes in the underlying network.

**Figure 2:**
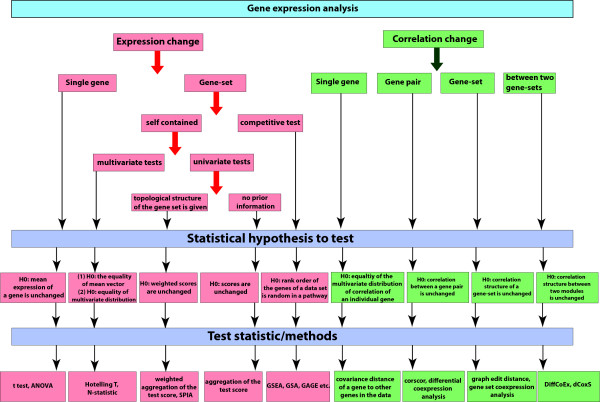
**Classification of different univariate and multivariate hypothesis testing based methods for gene expression data analysis.** The data are used to test differential expression and co-expression between two conditions for single gene and gene set based approaches.

In summary a gene expression data set can be used to (I) identify differentially altered single genes, (II) identify differentially expressed gene sets or pathways, and (III) identify differentially correlated pathways. In the following sections, we review statistical methods that have been introduced to study the three problems (I-III) above. In Figure 2 we give a graphical overview of the such methods.

Before we procede, we would like to point out that all of these methods test statistical hypotheses [46]. That implies that in order to understand a particular method biologically, i.e., one is capable of providing a biological interpretation, one needs to understand the underlying null hypothesis. In our opinion, it is helpful to approximately categorize all statistical hypotheses into three categories with respect to their biological interpretability, whereas each category represents a different degree of difficulty to find a biological interpretation for a hypothesis. In the following, we provide a brief discussion of these three categories because it enables a better, potentially, more plausible understanding of the methods presented in the next sections.

In category one belong all hypotheses for which it is relatively easy to find a meaningful biological interpretation. An example from this category are tests that compare mean values (*μ*), e.g., to identify the differential expression of genes (section ‘Differential expression of a gene’). That means these tests use the mean as a *test statistic*. Due to the fact that the underlying (probability) distribution of the genes represents, biologically, the activity of the gene expressions, the interpretation of a null hypothesis is directly derived thereof. For this reason the biological interpretation of the rejection of the null hypothesis given in Eqn. 2, is intuitively clear and appealing, because it implies a change in the (mean) expression of genes which may indicate a change in a biological function because the number of available proteins may be altered.

In category two fall tests for which there are several alternative biological interpretations. This makes the interpretation of such tests ambivalent from a biological perspective. As example for such a test, we consider the detection of the differential variance of a gene (section ‘Differential variance of a gene’). Despite the fact that the underlying probability distribution of the expression of genes has a clear biological interpretation, the biological interpretation for the rejection of the null hypothesis in Eqn. 4 is not unique. For instance, a gene could have a different variance in two conditions because, e.g., in condition one it is periodically expressed, whereas in condition two it is constantly expressed on an intermediate level. The former condition may be related to the cell cycle or the circadian rhythm, or periodically triggered by an external signaling factor that is released by the administration of a medication that is regularly taken. A second equally plausible interpretation could be that in one condition the cell utilizes parallel pathways to transfer a signal whereas in the other condition only one signaling chain is used. The reason for the utilization of parallel pathways could be triggered by stress factors, e.g., in the presence of an infection, so that the cell is ‘running’ full power in order to execute all necessary programs that have been initiated by the presence of the intruder.

Lastly, for tests in category three it is very difficult to find sufficiently precise biological interpretations because, statistically, these methods test ‘complex’ expressions. An example for a test from this category is the N-statistic (section ‘N-statistic’). The null hypothesis is based on the comparison of two *distributions* rather than *scalar* test statistics. In order to clarify the crucial difference between the comparison of two *distributions* and *scalar* test statistics we note that, theoretically, every probability distribution can be written as a series expansion in its moments [47]. This means a test for a distribution, compares *implicitly* the moments of this distribution. Here the (k-th) moment is defined as the expectation value of a random variable (to power k), i.e.,

(1) $$m_{k}=E[x^{k}].$$

An example for a moment is the mean (which is the first moment), other examples of entities that can be expressed as a function of moments are the variance and the kurtosis. This means whenever the null hypothesis in Eqn. 39 is rejected it could be because of a difference in *any* moment of which there are, theoretically, infinite many. Put differently, this kind of unspecificity makes this test very powerful in the sense that it may detect *any possible* difference two distributions can exhibit. On the other hand, if the null hypothesis is rejected it is very difficult to identify a precise reason for its rejection. For instance, this could be related to a difference in the mean, variance, kurtosis or any higher moment or function thereof. These combinatorial factors do usually not allow to find a concise biological interpretation. Nevertheless, such a test can be of valuable use, e.g., for diagnostic purposes.

## Single-gene analysis

Single-gene based methods can be subdivided into three major classes. A) Methods for detecting differential gene expression, B) methods for detecting differential correlation, and C) methods for detecting a differential variance.

### Differential expression of a gene

The analysis of differential gene expression is based on the mean expression change of individual genes. Suppose for a gene *g*_*i*_ in a microarray data set the mean expression value for two conditions are *μ*_1_ and *μ*_2_ respectively. Then the null hypothesis for the differential expression of the gene *g*_*i*_ is defined as

(2) $$H_{0}:\mu_{1}=\mu_{2}$$

(3) $$H_{1}:\mu_{1}\neq \mu_{2}$$

**Figure 3:**
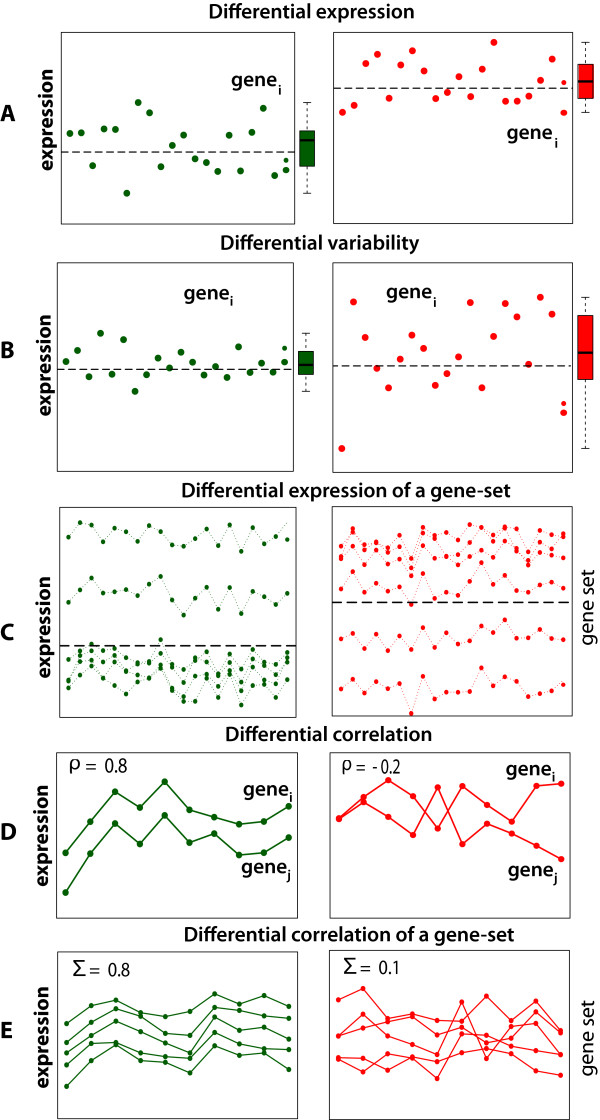
**Examples for different biological hypothesis that can be formulated based on different properties of gene expression data (green - control group (condition 1), red - treatment group (condition 2)).** E: *Σ*corresponds to the covariance matrix, corresponding to a constant but different matrix for the two conditions.

A gene is called *differentially expressed* when *H*_0_ is rejected. 3 Figure 3A shows an example where the samples for two conditions are drawn from two normal distributions with different mean values, i.e., *N*(*μ*_1_ = 0,*σ*_1_ = 1) and *N*(*μ*_2_ = 1,*σ*_2_ = 1).

The first published studies for gene expression analysis selected differentially expressed genes based on a fold-change criteria between a treatment and control condition [48]. For example, an early application of this measure was used to compare normal colon epithelium and primary colon cancers [49]. Since then, many statistical approaches have been developed to provide more robust measures. Among the most popular methods are, e.g., SAM [50,51], limma [52], and the empirical Bayes approach from Efron et al. [53].

### Differential variance of a gene

The analysis of *differential variability* (DV) aims to detect a change in the variance of the gene expression values [54]. Suppose in a microarray expression data set, the mean expression value of a gene *i* is *μ*_*c*_ and its variance $\left(\sigma_{c}^{2}\right)$, for condition *c* = {1,2}. Then, the null and alternative hypothesis tested are:

(4) $$H_{0}:\sigma_{1}^{2}=\sigma_{2}^{2}$$

(5) $$H_{1}:\sigma_{1}^{2}\neq \sigma_{2}^{2}$$

A gene is called *differentially variable* when the null hypothesis *H*_0_ is rejected. The DV analysis in [54] tests *H*_0_ by using a F-test. In Figure 3B we show an example for a gene with a constant mean, but a changed variance in the two conditions. The samples for the two conditions are drawn from a standard normal distribution with the same mean but different variances for the conditions, i.e., *N*(*μ*_1_ = 0,*σ*_1_ = 1), and *N*(*μ*_2_ = 0,*σ*_2_ = 2).

### Differential correlation of a gene

The analysis of *differential correlation* aims to detect changes in the dependency structure of a single gene [55]. Suppose *r*_*i*_ = *r*_*i*1_,…,*r*_*ip*_ denotes a *p* − 1 dimensional correlation vector, whereas each component corresponds to the correlation between gene *i* and one of the other *p* − 1 genes in a data set. Then for *r*_*i*_ one obtains distribution functions, denoted by $\left(F_{r_{i}}^{A}\right)$ and $\left(F_{r_{i}}^{B}\right)$, for condition *A* and *B* and the following hypotheses:

(6) $$H_{0}:F_{r_{i}}^{A}=F_{r_{i}}^{B}$$

(7) $$H_{1}:F_{r_{i}}^{A}\neq F_{r_{i}}^{B}$$

A gene *i* is called *differentially correlated* when *H*_0_ is rejected.

## Gene-pair analysis

The functional activities of genes, as measured by gene expression values, reflect the interplay of the genes and their products in the underlying gene network. The objective of a gene-pair analysis is to identify either differential co-correlated or differential co-expressed pairs of genes, instead of individual genes. The reason for looking for pairs of genes is that the concerted changes in genes is due to their common membership in biological pathways.

The principle idea to detect correlation changes in gene-pairs is visualized in Figure 3D. The data are sampled from a multivariate normal distribution with a constant mean vector for both conditions, ***μ***_**1**_ = ***μ***_**2**_ = (0,1), but a different correlation of *ρ*_1_ = 0.8 and *ρ*_1_ = −0.2. The point is despite no difference in the mean expression of the gene-pair, there is a difference in their correlation.

**Figure 4:**
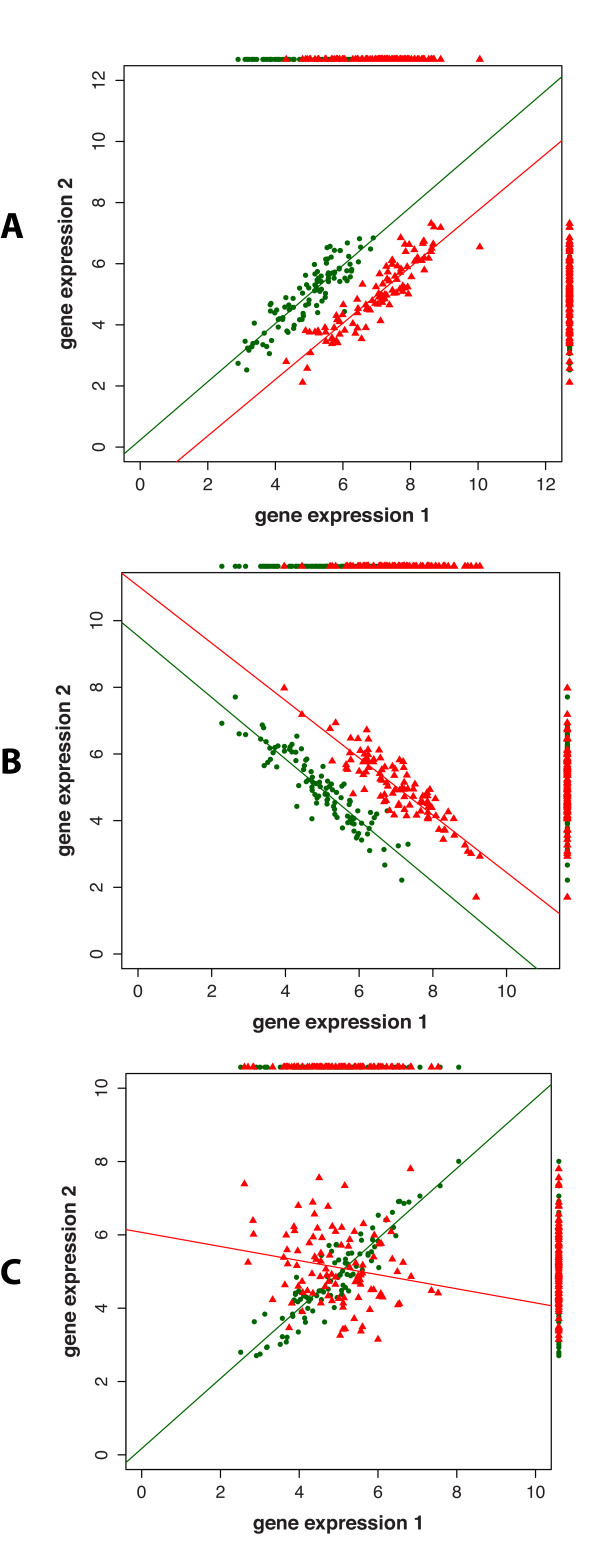
**The joint distribution of the expression of two genes.** In **A** and **B** the expression values of the two genes show a shift in the two conditions (red and green). In **C** the expression values of a gene pair are anti-correlated between the two conditions. In **A-C** the lines correspond to linear regression fits of the colored expression values and the color dots surrounding the figures provide one-dimensional projections of the two-dimensional distributions.

In [56] a method (CorScor) has been proposed to identify such gene-pairs. In Figure 4 we show three cases of the joint distribution of expression values of two genes, for two conditions. In this Figure we are showing simulated data for three possible changes in the co-expression of a pair of genes in two conditions. The samples in Figure 4A and B are drawn from a multivariate normal distribution with ***μ***_1_ = {5,5} and ***μ***_2_ = {5,7}. For Figure A the correlation between gene-pairs is *ρ*_1_ = *ρ*_2_ = 0.9 and for Figure B it is *ρ*_1_ = *ρ*_2_ = −0.9. For Figure 4C the samples are generated from a multivariate normal distribution with ***μ***_1_ = {5,5} and ***μ***_2_ = {5,5} and the average correlation between gene-pairs is *ρ*_1_ = 0.9 and *ρ*_2_ = −0.3.

In the first two cases (Figure 4A and B) the correlation of the gene-pairs show a condition specific shift, in [56] denoted as a *gap* and *substitution*. In the third case (Figure 4C), the gene-pairs show a reversed correlation between the two conditions, denoted as *on/off case*. To identify gene-pairs in these two types of conditions, two scoring functions have been suggested in [56] given by:

(8) $$s=\left\{\begin{array}{cc} \left|\rho_{A}+\rho_{B}-\alpha \rho \right| & \;\text{gap/substitution case}\\ |\rho_{A}-\rho_{B}|\; & \text{on/off case}\;\; \end{array}\right)$$

Here each of the three correlation coefficients are estimated for a gene-pair between gene *i* and *j*, i.e., *ρ*_*A*_ = *ρ*_*A*_(*ij*) etc. The value of *ρ* corresponds to the global correlation coefficient of the gene-pair over the two conditions (*AB*) and *ρ*_*A*_, *ρ*_*B*_ are the correlation coefficients of the gene-pair for condition *A* and condition *B*. In Eqn. 8, *α* is a tuning parameter that governs the balance between separation and parallel alignment. In [56] it was argued to use a value of *α* = 1.5. The null and alternative hypotheses tested are:

(9) $$H_{0}:s=0$$

(10) $$H_{1}:s\neq 0$$

In [57] the ‘expected conditional F-statistic’ (ECF-statistic) has been introduced to measure the differential co-expression of gene pairs (*XY*). The method is based on a modified F-statistic, where the variance and the mean parameter of the statistic are estimated from a mixture of two normal distributions.

The R package R/EBcoexpress provides an empirical Bayesian implementation to identify the differential co-expression of gene pairs [58].

Another method called *liquid association* (LA) has been proposed in [59] to identify co-expressed gene pairs. In contrast to pairwise correlation measures, the LA method considers the presence of a mediator gene, *Z*, for observing a co-expression between two genes at a given cellular state. Let *X*, *Y * and *Z* be gene-expression profiles. We say that *X* and *Y * form a *liquid association pair* (LAP), if the cellular state of *X* and *Y * is correlated with *Z*. The LA score of *X* and *Y * with respect to *Z* is estimated from rank transformed expression profiles of *X*, *Y*, *Z* given by

(11) $$\mathrm{LA}(X,Y|Z)=\frac{1}{m}\overset{m}{\underset{i=1}{\sum }}X_{i}Y_{i}Z_{i}$$

Here *m* corresponds to the number of samples. The LA method uses a permutation test for the identification of significant LA gene pair values. Due to the high computational burden of the method that would require *N*^3^ (N is the number of genes) evaluations of Eqn 11 plus additional permutations of the data, which is even for only *N* = 10^3^ genes intractable because it requires already more than 10^9^ evaluations. For this reason the method is only used to (A) find the gene *Z* for a given pair of genes or (B) find the LAP, *X* and *Y*, for a given gene *Z*.

## Gene set and pathway analysis methods

Generally, a pathway is a group of interacting genes (a gene set) that deploy a cellular function. In a biological system the biological processes are coordinated functions of sets of genes which make the organism work. Some general pathways are, e.g., metabolic pathways, signaling pathways or regulation pathways that represent minimal functioning units of a cellular system. The consideration of pathways or gene sets for a comparative gene expression analysis is an important step toward the exploration of relevant functional mechanisms of a cell.

So far, many multivariate and univariate tests have been proposed for a gene set analysis, see Figure 2. Finding differentially expressed pathways, instead of individual genes, is not straight forward from a statistical and biological perspective and there are several hurdles to this approach. The first is presented by the data themselves, because the number of variables is usually (much) larger than the number of samples, i.e., *n* << *p*, that leads to many estimation problems. The second hurdle is our incomplete information about the constitution of biological pathways and the potentially high overlap of genes between different pathways. For example, databases like GO [60] provide valuable information about genes for a large variety of different organisms. However, this information is not static but continuously expanding leaving us at the moment with a snap-shot of knowledge. This makes it difficult to find precise definitions for particular pathways of interest. The third problem comes from the underlying gene network structure that describes the true interactions between genes in a pathway. Here, the problem is that as a result of such interactions among genes it is usually not appropriate to assume their independence, as frequently done for statistical ease.

A motivating example for the general idea underlying gene set methods is shown in Figure 3C. For condition 1 (green), the samples are drawn from a multivariate normal distribution with ***μ***_1_ = {2,2.2,2.4,2.6,5,8} and for condition 2 (red) ***μ***_2_ = {2,7,7.5,2.6,5,8}. The covariance matrix, ***Σ***_1_ = ***Σ***_2_ is for both conditions the same. In this Figure, only 2 of the 6 gene are differentially expressed. This reflects biological situations because, usually, only of fraction of the genes belonging to a pathway is found to be differentially expressed. However, due to the fact that gene set methods are based on the expression of a set of genes such methods borrow strength from the combined analysis of the genes.

Reviews that focus entirely on gene set and pathway analysis methods can be found in [61-65].

### Null hypothesis for gene set analysis

Gene set analysis methods can be broadly divided into two major categories, depending on what null hypothesis is tested. The first type of methods are called *competitive methods*, and the second type *self-contained methods*[66]. Briefly, self-contained tests use only the data from a target gene set under investigation, whereas competitive tests use, in addition, also data *outside* the target gene set (background data). In the following we describe popular competitive and self-contained pathway methods.

## Competitive gene set and pathway methods

In Table 1 we show an overview of gene set and pathway methods, described in the following.

**Table 1:** Overview of different competitive gene set methods

**Principle Method**	**Reference**	**Test type**	**Software**
Over-representation analysis (hypergeometric test)	[67]	parametric	GOstats
GSEA	[68]	non-parametric	GSEABase
	[27]	non-parametric	http://www.broad.mit.edu/gsea
GSA	[69]	non-parametric	http://cran.r-project.org/web/packages/GSA/
GSEArot	[70]	non-parametric	limma
GAGE	[71]	parametric	GAGE
PAGE	[72]	parametric	PGSEA, GAGE
Random Set	[73]	parametric	part of CLEAN
Generalized Random Sets	[74]	parametric	http://GenomicsPortals.org
Gene set enrichment analysis made simple	[75]	parametric	

If available, the name of the software package is provided.

### GSEA

The *gene set enrichment analysis* method (GSEA) [27,68] is one of the most widely used competitive test based method. The test uses a Kolmogorov-Smirnov test statistic to identify differential expressed gene sets. The gene set and background data set are defined in the following. Let *W * be the target gene set to be tested and *W*^*c*^ its complement in a way that the union of both sets defines all genes, i.e., *V* = *W* ∪ *W*^*c*^, in the data set. The hypotheses tested by GSEA with respect to an *enrichment score* (ES) are:

$$\begin{array}{c} H_{0}:\mathrm{ES}=0(\mathrm{vanishingtestscore})\;\\ H_{1}:\mathrm{ES}\neq 0(\mathrm{non}-\mathrm{vanishingtestscore}) \end{array}$$

Briefly, GSEA consists of the following steps, applied to each pathway:

(1) Estimation of gene-level test statistics.

(2) Rank ordering of the test statistics.

(3) Calculation of an enrichment score (ES) for a pathway based on the gene-level test statistics.

(4) Permutation of the gene-labels to estimate the significance of the enrichment score for the pathway.

### GSEArot

GSEArot (gene set enrichment analysis rotation) [70] is very similar to GSEA, but uses a different approach to randomize data in order to assess the significance of a target pathway. More specifically, a data matrix ***X*** is randomized by, first, rotating ***X*** around a random angle *δ*, resulting in a matrix ***X***(*δ*). Second, from the matrix ***X***(*δ*), the randomization matrix is obtained by a QR decomposition [76]. In [70] it is argued that this procedure has an advantage for small sample sizes, when only very few permutations are achievable from sample-label permutations. The null hypothesis tested by GSEArot is the same as for GSEA.

### Random set

The *random set* method introduced in [73] is a parametric test that is a generalization of Fisher’s exact test in the sense that enrichment scores of gene sets are compared with randomly formed sets. The enrichment scores are based on single gene-level test statistics reflecting their differential expression.

1. Estimate the enrichment score of a target gene set *W*,

(12) $$\bar{s}=\frac{1}{m}\underset{i\in W}{\sum }s_{i}.$$

Here *s*_*i*_ are gene-level scores, e.g., t-scores, and *m* = |*W*| is the number of genes in the target pathway.

2. Estimate the enrichment score and its variance of the background gene set *V* = *W* ∪ *W*^*c*^,

(13) $$\mu =\frac{1}{p}\underset{i\in V}{\sum }s_{i},$$

(14) $$\sigma^{2}=\frac{1}{m}\left(\frac{p-m}{p-1}\right)\left(\frac{\underset{i\in V}{\sum }s_{i}^{2}}{p}-{\left(\frac{\underset{i\in V}{\sum }s_{i}}{p}\right)}^{2}\right),$$

with *p* = |*W* ∪ *W*^*c*^|.

3. Estimate the standardized enrichment score

(15) $$Z=\frac{\bar{s}-\mu }{\sigma }.$$

The *Z* score follows a standard normal distribution under the null hypothesis *H*_0_ given by:

(16) $$\begin{array}{c} H_{0}:\text{The target set}\;W\;\text{is not}\;\text{enriched for differentially}\\ \text{expressed genescompared with}\;W\cup W^{c}\; \end{array}$$

(17) $$\begin{array}{c} H_{1}:\text{The target set}\;W\;\text{is}\;\text{enriched for differentially}\\ \;\text{expressed genescompared with}\;W\cup W^{c} \end{array}$$

It is notable that *Z* can be calculated without a numerical randomization of the data. Further, the background data consist of all genes *V*, including the ones in the target pathway *W*. In [77] this method has been applied to *head and neck* and *cervical cancer* for human papillomaviruses-positive and -negative samples.

### GAGE

GAGE [71] (generally applicable gene set enrichment) is also a parametric test that, similarly to GSEA, compares the expression in a target gene set with that of the background. But instead of using a Kolmogorov-Smirnov like test [78] it employes a two-sample t-test. The principle steps of the method are as follows:

1. Estimate the mean fold change *f* and its standard deviation *σ*_*f*_ for the *m* genes in the target pathway *W*.

2. Estimate the mean fold change *f*^*′*^and its standard deviation $\left(\sigma_{f^{\prime }}\right)$ for all *p* genes in the background gene set *V* = *W* ∪ *W*^*c*^.

3. Estimate the t-score:

(18) $$t=\frac{f-f^{\prime }}{\sqrt{\sigma_{f}^{2}/m+\sigma_{f^{\prime }}^{2}/m}}$$

with

(19) $$\mathrm{df}=(m-1)\frac{{\left(\sigma_{f}^{2}+\sigma_{f^{\prime }}^{2}\right)}^{2}}{\sigma_{f}^{4}+\sigma_{f^{\prime }}^{4}}$$

degrees of freedom.

Also GAGE employs all genes *V * in the background gene set, including the ones in the target set *W*. The underlying assumption of GAGE is that the (mean) fold changes of genes are independent and identically distributed. The null hypothesis tested by GAGE is:

(20) $$\begin{array}{c} H_{0}:\text{The mean fold change of genes (MFG) in set}\\ W\;\text{is not}\;\text{differentto the MFG in}\;W\cup W^{c} \end{array}$$

(21) $$\begin{array}{c} H_{1}:\text{The mean fold change of genes (MFG) in set}\\ W\;\text{is}\;\text{different tothe MFG in}\;W\cup W^{c}\; \end{array}$$

### GSA

Another method is GSA (gene set analysis) [69]. The method, first, calculates z-scores, *z*_*i*_, with *i* ∈ {1,…,*m*}, for all *m* genes in a given target pathway *W*. Then each z-score is transformed into two scores, assessing the sign of *z*_*i*_.

(22) $$s^{+}(z)=\max\{z,0\}$$

(23) $$s^{-}(z)=-\min\{z,0\}$$

This results in two sets of non-zero scores $S^{+}=\{s^{+}(z_{1}),\ldots ,s^{+}(z_{m})\}$ and $S^{-}=\{s^{-}(z_{1}),\ldots ,s^{-}(z_{m})\}$ from which their mean value is calculated,

(24) $${\bar{s}}^{+}=\mathrm{mean}(S^{+})=\frac{1}{m}\underset{i}{\sum }s^{+}(z_{i})$$

(25) $${\bar{s}}^{-}=\mathrm{mean}(S^{-})=\frac{1}{m}\underset{i}{\sum }s^{-}(z_{i})$$

Finally, the *maxmean* test statistic is defined by $s_{\mathrm{mm}}=\max\{{\bar{s}}^{+},{\bar{s}}^{-}\}$, giving the test statistic for the target pathway.

(26) $$H_{0}:s_{\mathrm{mm}}(W)=s_{\mathrm{mm}}(W^{c})$$

(27) $$H_{1}:s_{\mathrm{mm}}(W)\neq s_{\mathrm{mm}}(W^{c})$$

The null distribution is assessed by a *restandardization*, combining a sample- and gene-label permutation.

## Self-contained gene set and pathway methods

In Table 2 we show an overview of self-contained gene set and pathway methods that are describe in the following in more detail.

**Table 2:** Overview of self-contained gene set and pathway methods

**Principle Method**	**Reference**	**Software**
Average of single-gene statistics	[79]	sigPathway
Linear Model Toolset for GSEA	[80]	GSEAlm
SAM-GS	[81]	
Globaltest	[82]	globaltest
GlobalANCOVA	[83]	GlobalAncova
Hotelling’s *T*^2^	[84-87]	PCOT2
N-statistic	[88]	cramer
RCMAT	[89]	
Non-linear tests for identifying differentially expressed genes or genetic networks	[87]	
Pathway-express	[90]	
Signaling Pathway Impact Analysis	[91]	SPIA (Bioconductor)
SEPEA	[92]	
PARADIGM	[93]	
Gene set analysis exploiting the topology of a pathway	[94]	IPS (available upon request)

### Sum of t-square

The *sum of t-square* test is an univariate test that is based on t-scores, {*t*_*i*_}, individually obtained for each of the *m* genes in a given set [95], see also [79]. That means that each t-score assesses the difference of the mean expression between the two conditions,

(28) $$t_{i}=\frac{\Delta {\bar{x}}_{i}-\Delta \mu_{i}}{{\bar{s}}_{i}}$$

with *s*_*i*_ the pooled standard deviation. The test statistic for a pathway is based on the individual t-scores given by

(29) $$\mathrm{TS}=\frac{1}{m}\sqrt{\overset{m}{\underset{i}{\sum }}t_{i}^{2}}.$$

Because for each gene *Δμ*_*i*_ = 0 should hold if a gene is not differentially expressed, the null and alternative hypothesis can be formulated as:

(30) $$H_{0}:\mathrm{TS}=0\text{(vanishing test score)}$$

(31) $$H_{1}:\mathrm{TS}\neq 0\text{(non-vanishing test score)}$$

The significance of TS is assessed from sample-label permuted data.

### SAM-GS

The method *SAM-GS* (Significance Analysis of Microarray for gene sets) [81] uses the test statistics,

(32) $$\text{SAM-GS}=\overset{m}{\underset{k=1}{\sum }}d_{k}^{2},$$

with $d_{k}=\frac{{\bar{x}}_{1k}-{\bar{x}}_{2k}}{s_{k}+s_{0}}$. Here $\left({\bar{x}}_{1k}\right)$ and $\left({\bar{x}}_{2k}\right)$ are the sample means of the control and treatment condition of gene *k*, *s*_*k*_ corresponds to its pooled standard deviation and *s*_0_ is a constant for a sensitivity adjustment. The null and alternative hypothesis can be formulated as:

(33) $$H_{0}:\text{SAM-GS}=0\text{(vanishing test score)}$$

(34) $$H_{1}:\text{SAM-GS}\neq 0\text{(non-vanishing test score)}$$

Statistical significance of *SAM-GS* is again assessed from sample-label permuted data.

### Hotelling’s *T*^2^

The Hotelling *T*^2^ test is a self-contained test that is a multivariate generalization of the univariate t-test. Its null and alternative hypothesis can be formulated as:

(35) $$\begin{array}{c} H_{0}:\mu^{T}=\mu^{C}\text{(equality in the m-dimensional}\\ \;\text{population mean vectors)} \end{array}$$

(36) $$\begin{array}{c} H_{1}:\mu^{T}\neq \mu^{C}\text{(difference in the m-dimensional}\\ \;\text{population mean vectors)} \end{array}$$

Suppose we have two groups with *n*_*C*_ samples from the control group and *n*_*T*_ samples for the treatment group, each consisting of *m* genes. Let the expression level of the *i*^*th*^ sample of the control group and treatment group be given by $X_{i}^{C}={\left(X_{i1}^{C},X_{i2}^{C},\ldots X_{\mathrm{im}}^{C}\right)}^{t}$ and $X_{i}^{T}={\left(X_{i1}^{T},X_{i2}^{T},\ldots X_{\mathrm{im}}^{T}\right)}^{t}$, respectively. The pooled covariance matrix **S**is then defined by

(37) $$\text{S}=\frac{(n_{T}-1){\text{S}}_{T}+(n_{C}-1){\text{S}}_{C}}{\left(n_{T}+n_{C}-2\right)}$$

where **S**_*C*_ and **S**_*T*_ are the covariance matrices for the control and treatment group. Hotelling’s *T*^2^ is defined as

(38) $$T^{2}=\frac{n_{T}\times n_{C}}{n_{T}+n_{C}}\left(\mu^{T}-\mu^{C}\right){\text{S}}^{-1}{\left(\mu^{T}-\mu^{C}\right)}^{t}.$$

The inverse of the covariance matrix is estimated via the shrinkage estimator [96-99]. The statistical significance of the test statistic *T*^2^ is estimated from sample-label permuted data.

### N-statistic

The N-statistic is a non-parametric test that is used to test the equality of two distributions. Suppose the expression level of the *i*^*th*^ sample of the control group, *n*_*C*_, and the treatment group, *n*_*T*_, is given by $X_{i}^{C}={\left(X_{i1}^{C},X_{i2}^{C},\ldots X_{\mathrm{im}}^{C}\right)}^{t}$ and $X_{i}^{T}={\left(X_{i1}^{T},X_{i2}^{T},\ldots X_{\mathrm{im}}^{T}\right)}^{t}$, respectively. Let *i* ∈ {1…*n*_*C*_} correspond to the control data-set and *i* ∈ {1…*n*_*T*_} to the treatment data-set. The null and alternative hypothesis tested by the N-statistic can be formulated as:

(39) $$H_{0}:F_{C}(x)=F_{T}(x)$$

(40) $$H_{1}:F_{C}(x)\neq F_{T}(x)$$

whereas *F*_*C*_(*x*) and *F*_*T*_(*x*) are two multivariate distribution functions from the control and the treatment condition.

The N-statistic itself is defined as follows:

(41) $$\begin{array}{ccc} \hat{N} & = & \left[\frac{1}{n_{C}n_{T}}\overset{n_{C}}{\underset{i=1}{\sum }}\overset{n_{T}}{\underset{j=1}{\sum }}2K\left(x_{i}^{C},x_{j}^{T}\right)-\frac{1}{n_{C}^{2}}\overset{n_{C}}{\underset{i=1}{\sum }}\overset{n_{C}}{\underset{j=1}{\sum }}K\left(x_{i}^{C},x_{j}^{C}\right)\right)\\ & & {\left(-\frac{1}{n_{T}^{2}}\overset{n_{T}}{\underset{i=1}{\sum }}\overset{n_{T}}{\underset{j=1}{\sum }}K\left(x_{i}^{T},x_{j}^{T}\right)\right]}^{1/2} \end{array}$$

Here $K\left(x_{i}^{C},x_{j}^{T}\right)$, defined as $K\left(x_{i}^{T},x_{j}^{C}\right)={∥x_{i}^{T}-x_{j}^{C}∥}_{2}$, is the Euclidean Kernel serving as distance function between the expression values in the two conditions.

### Linear model-based pathway methods

There are also several approaches that utilize either a linear or a generalized linear modeling framework for a gene set analysis. Examples for such methods are Global test [82], Extension of GSEA [80] or GlobalAncova [83].

### Topological pathway methods based on existing network information

Some recent univariate methods, for instance, Pathway-express [90], SPIA [91] or SEPEA [92], use instead of correlation measures to estimate interactions among genes, predefined topological information as provided, e.g., by the KEGG database [100]. These methods assign each gene in a pathway a score that is based on the position of a gene in the given network structure and, finally, aggregate these individual gene scores to obtain a score for the pathway itself. Yet another approach is provided by PARADIGM [93]. This method uses a factor graph model combining gene copy number variation data with gene expression data for the identification of differentially expressed pathways.

### Iterative Proportional Scaling: IPS

IPS (Iterative Proportional Scaling) [94,101] is another method that utilizes the topology of pathways of a given gene set by testing the hypotheses:

(42) $$H_{0}:\Sigma_{c_{1}}^{-1}=\Sigma_{c_{2}}^{-1}\;\;\text{:assuming}\Sigma_{c_{1}}^{-1},\Sigma_{c_{2}}^{-1}\in S^{+}(G)$$

(43) $$H_{1}:\Sigma_{c_{1}}^{-1}\neq \Sigma_{c_{2}}^{-1}\;\;\text{:assuming}\Sigma_{c_{1}}^{-1},\Sigma_{c_{2}}^{-1}\in S^{+}(G)$$

In this method, the covariance matrices, $\left(\Sigma_{c_{1}},\Sigma_{c_{2}}\right)$, are estimated from the data, for both conditions, using the Iterative Proportional Scaling (IPS) algorithm. The inverse of the estimated covariance matrices are positive definite (concentration) matrices for which it is assumed that the non-zero elements in $\left(\Sigma_{c_{1}}^{-1}\right)$ and $\left(\Sigma_{c_{2}}^{-1}\right)$ are identical; this is the meaning of $\Sigma_{c_{1}}^{-1},\Sigma_{c_{2}}^{-1}\in S^{+}(G)$ where *S*^ + ^indicates the class of all symmetric positive definite matrices with non-zeros elements given by the binary matrix *G*. This means that the concentration matrices, $\Sigma_{c_{1}}^{-1}$ and $\Sigma_{c_{2}}^{-1}$, have identical zero element, but are allowed to have different non-zero entries. In other words, it is assumed that the underlying topology of a pathway is the same for condition *c*_1_ and *c*_2_, given by *G*, whereas *G*_*ij*_ = 0 corresponds to an ‘absent’ interactions among the genes *i* and *j*. Since the structure of *G* is not estimated from the data, it is necessary to obtain it from an independent source, e.g., from the KEGG database or Reactome [100,102]. In [94] it is shown that a log likelihood (log(*Λ*)) ratio test can be used to test for the equality of the concentration matrices for the two conditions and that asymptotically the log likelihood ratio follows a Chi-square distribution with *r* + *m* degrees of freedom, i.e., $\log(\Lambda )∼\chi_{r+m}^{2}$, whereas *m* is the number of genes in the pathway and *r* is the number of non-vanishing edges in *G* corresponding to the fixed interaction structure of the pathway.

The IPS method has been used in [94] to study acute lymphocytic leukemia with and without BCR/ABL gene rearrangement. As a result, the JUN oncogene with RAS/MAPK/JNK followed by NFAT and NFKB seem to be crucial in distinguishing BCR/ABL positive and negative patients.

## Differential correlation/interaction methods

In the previous sections, we discussed different gene set and pathway-based methods for the identification of differentially expressed pathways. These methods focused either only on the expression of genes, or considered an underlying interaction topology among the genes as taken from an independent source. However, even when these methods considered an underlying network structure, this structure was assumed to be the same for the ‘treatment’ and ‘control’ group.

In contrast, in this section we discuss methods that *estimate* the correlation/interaction structure of the genes within pathways, for each experimental condition. The underlying rationale for these approaches is to assume that the expression profiles of genes are dependent on each other [103,104] as the genes in a pathway interact, either directly or indirectly [105]. This assumption results from the observation that genes with similar functions or cellular localization are often co-expressed and cluster together. The methods discussed in this section bear a similarity to the statistical methods for the estimation of differential correlated gene-pairs (see section ‘Gene-pair analysis’). However, the extension of such gene-pair measures to the pathway level allows the identification of pathways that show, e.g., a condition specific correlation change.

In Figure 3E we show a simulated example scenario for condition specific correlation changes of the expression profiles for a gene set. In Figure 3E the correlation between all gene-pairs of a gene set is aggregated by a summary statistic. In this example, the mean values between the genes is of a comparable order, whereas the correlation of the gene set in the treatment condition is reduced.

A variety of different pathway methods have been developed that integrate the estimated gene correlations or co-expression structures with gene expression data. A summary of different methods that are used for the identification of differential correlation/interaction changes in pathways is shown in Table 3. In the following these methods are described in more detail.

**Table 3:** Overview of methods for the identification of differential correlation/interaction changes in pathways

**Principle Method**	**Reference**	**Software**
Graph edit distance	[106]	
Gene-set co-expression analysis (GSCA)	[107]	GSCA (http://www.biostat.wisc.edu/∼kendzior/GSCA/)
Differential co-expression (dCoxS) between gene-sets	[108]	dCoxS (http://www.snubi.org/publication/dCoxS/index.html)
DiffCoEx	[109]	R code is provided in the paper
Differential disease network using C3NET	[106,110]	c3net (http://cran.r-project.org/web/packages/c3net/index.html)
Disease associated interactions using Synergy network	[111]	MATLAB code is provided in the paper

### Graph Edit Distance: GED

Among the first approaches that estimate the interaction structure for a pathway analysis to identify *differentially correlated pathways* (DCP) is a method introduced in [106]. This method uses the *graph edit distance* (GED) score as a test statistic.

More precisely, for a given pathway containing *m* genes an association graph, *G*, also called *pseudo-pathway*, is inferred for each condition. That means the resulting network comprises the *m* genes of this pathway only. The inference method estimates correlation and partial correlation coefficients and tests their statistical significance. That means, if either the correlation or partial correlation coefficient for two genes *i* and *j* in this pathway vanishes, then the resulting network will *not* have an interaction between gene *i* and *j*, i.e., *E*_*ij*_ = 0, otherwise there is an interaction, *E*_*ij*_ = 1. Here *E* corresponds to the adjacency matrix of the network. Suppose $\left(G_{c_{1}}\right)$ and $\left(G_{c_{2}}\right)$ are two networks that have been inferred from gene expression data for condition *c*_1_ and *c*_2_. Further, assume that *M*_1_,*M*_2_,…,*M*_*n*_ are all possible transformations that map $\left(G_{c_{1}}\right)$ into $\left(G_{c_{2}}\right)$, i.e, $\left(M_{i}(G_{c_{1}})=G_{c_{2}}\right)$. Then the *optimal cost* of the optimal transformation, *M*^*′*^, is given by *c*(*M*^*′*^) = min{*c*(*M*_*i*_)|1 ≤ *i* ≤ *n*}. This value is used to define a dissimilarity measure $d_{\text{GED}}(G_{c_{1}},G_{c_{2}})=c(M^{{}^{\prime }})$ between the two networks $\left(G_{c_{1}}\right)$ and $\left(G_{c_{2}}\right)$, called the *graph edit distance* (GED) score [112]. For arbitrary networks, $\left(G_{c_{1}}\right)$ and $\left(G_{c_{2}}\right)$, the estimation of *d*_GED_(*G*_*A*_*G*_*B*_) is numerically challenging. However, for our specific problem it can be efficiently calculated based on the adjacency matrices, $\left(E^{c_{1}}\right)$ and $\left(E^{c_{2}}\right)$, of the two networks,

(44) $$d_{\text{GED}}(G_{c_{1}},G_{c_{2}})=\frac{2}{m(m-1)}\overset{m}{\underset{\text{ij}}{\sum }}\left|E_{\text{ij}}^{c_{1}}-E_{\text{ij}}^{c_{2}}\right|.$$

In [106] the GED score has been used as a test-statistic for the formulation of the hypotheses:

(45) $$H_{0}:d_{\text{GED}}(G_{c_{1}},G_{c_{2}})=0$$

(46) $$H_{1}:d_{\text{GED}}(G_{c_{1}},G_{c_{2}})\neq 0$$

In order to assess statistical significance, sample label permutations are performed to obtain the null distribution.

Extensions of this method can be found in [113] where mutual information values have been used to capture non-linear relations among gene expression values. Further, in [114] a methods based on a *relevance value* (RV) has been defined for integrating different types of genomics data sets which has also a resemblance to the GED.

### Gene set co-expression analysis: GSCA

A method that is based on (zero-order) correlation coefficients is *gene set co-expression analysis* (GSCA) [107]. This method uses as test statistic the *dispersion index*, which is defined as follows:

(47) $$D_{s}(\rho^{c_{1}},\rho^{c_{2}})=\sqrt{\frac{1}{P}\overset{P}{\underset{k=1}{\sum }}{\left(\rho_{k}^{c_{1}},-,\rho_{k}^{c_{2}}\right)}^{2}}$$

Here $\left(\rho_{k}^{c}\right)$, with *c* ∈ {*c*_1_,*c*_2_}, is the k-th correlation coefficient for a gene pair, which can be formed among the total number, P=m2, of such pairs for a pathway consisting of *m* genes. The null and alternative hypotheses tested are:

(48) $$H_{0}:D_{s}(\rho^{c_{1}},\rho^{c_{2}})=0$$

(49) $$H_{1}:D_{s}(\rho^{c_{1}},\rho^{c_{2}})\neq 0$$

From the definition of the dispersion index follows that also this method aims at detecting at differential correlation among pathways, despite its name emphasizing co-expression. Interestingly, the *dispersion index* corresponds to the GED score if its components in Eqn. 47 are re-labeled and one defines the components of the adjacency matrices $\left(E^{c_{1}},E^{c_{2}}\right)$ as the correlation coefficients rather than the outcome of the hypotheses tests [106].

A visualization of the underlying idea of GSCA is shown in Figure 3E. The gene expression values are sampled from multivariate normal distribution *N*(***μ***_**1**_,***Σ***_1_) and *N*(***μ***_2_,***Σ***_2_) with ***μ***_1_ = ***μ***_2_, and the average covariance between gene-pairs is ***Σ***_1_ = 0.8 and ***Σ***_2_ = −0.2. Despite the fact that there is neither a difference in the individual expression of genes nor the the expression of a set of genes, condition 1 and 2 can be distinguished by using a measure based on a correlation change.

### Partial least squares based scores: PLS

A statistical framework based on a *partial least squares* score is proposed in [115]. Similar to the above methods, two matrices for the two conditions are inferred. These matrices can be seen as weighted networks, whereas an edge weight corresponds to the strength of the association between two genes. In this paper, three different types of tests are introduced that allow (A) testing for changes in the module structure of the two networks, (B) testing for changed in the connectivity of a particular gene set, and (C) testing for changes in the connectivity of a particular gene.

### Differentially co-expressed gene sets: dCoxS

In [108] the *differentially co-expressed gene sets* (dCoxS) algorithm is proposed. This is an entropy-based method that uses the *interaction score* (IS) to measure the difference between two pathways. The IS is estimated by the correlation coefficient between the entropies of the two pathways. The entropies themselves are estimated by using the *Rényi relative entropy*, which is defined by:

(50) $$\;D_{\alpha }(P∥Q)\;=\;\frac{1}{\alpha -1}\log\left(\int \left(p^{\alpha }q^{1-\alpha }\right)\mathrm{dp}\;\mathrm{dq}\right)\;\approx \;\log\frac{{\hat{f}}_{h}(S_{i})}{{\hat{f}}_{h}(S_{j})}$$

Here *α* is a parameter and $\left({\hat{f}}_{h}(S_{i})\right)$ and $\left({\hat{f}}_{h}(S_{j})\right)$ are expression densities of the samples *i* and *j*, estimated by using a multiplicative kernel for the density estimation. Further, *p* and *q* are the probability density functions of *P* and *Q*. From this, the *IS* is estimated by:

(51) $$\text{IS}=\frac{\sum_{i<j}(RE^{P_{1}}-\bar{R}E^{P_{2}})(RE^{P_{1}}-\bar{R}E^{P_{2}})}{\sqrt{\sum_{i<j}{\left(R,E^{P_{1}},-,\bar{R},E^{P_{1}}\right)}^{2}}\sqrt{\sum_{i<j}{\left(R,E^{P_{2}},-,\bar{R},E^{P_{2}}\right)}^{2}}}.$$

In this equation, $\left(RE^{P_{1}}\right)$ and $\left(RE^{P_{2}}\right)$ are entropy matrices of two gene sets, *P*_1_ and *P*_2_, for condition *c*_*i*_, whereas each component of the entropy matrices is proportional to $\left(∼\log\frac{{\hat{f}}_{h}(S_{i})}{{\hat{f}}_{h}(S_{j})}\right)$. That means, strictly *IS* = *IS*(*P*_1_(*c*_*i*_),*P*_2_(*c*_*i*_)). Application of Fisher’s Z-transformation to *IS* results in a z-score, *z*(*IS*(*P*_1_(*c*_*i*_),*P*_2_(*c*_*i*_))), for condition *c*_*i*_. Combination of both z-scores for condition *c*_1_ and *c*_2_ leads to,

(52) $$\begin{array}{c} z_{\mathrm{comb}}=\frac{z\left(\mathrm{IS},\left(P_{1},(,c_{1},),,,P_{2},(,c_{1},)\right)\right)-z\left(\mathrm{IS},\left(P_{1},(,c_{2},),,,P_{2},(,c_{2},)\right)\right)}{\sqrt{1/(n_{1}-3)+1/(n_{2}-3)}}. \end{array}$$

Here *n*_1_ and *n*_2_ correspond to the number of samples for condition *c*_1_and *c*_2_. The interpretation of the null hypothesis tested can be stated as:

(53) $$\begin{array}{c} H_{0}:z_{\mathrm{comb}}=0\left(\text{equality in entropy changes between}\right)\\ \;\;\;\;\text{gene-pairs in the pathways}\;P_{1}\;\text{and}\;P_{2}\\ \;\;\;\;\left(\text{between the conditions}c_{1}\;\text{and}\;c_{2}\right) \end{array}$$

(54) $$\begin{array}{c} H_{1}:z_{\mathrm{comb}}\neq 0\left(\text{difference in entropy changes between}\right)\\ \;\;\;\;\text{gene-pairs in the pathways}\;P_{1}\;\text{and}\;P_{2}\\ \left(\;\;\;\;\text{between the conditions}\;c_{1}\;\text{and}\;c_{2}\right) \end{array}$$

In [108] dCoxS has been applied to gene expression data from lung cancer. Their analysis identified the *Thrombin signaling and protease-activated receptors pathway*, which is known to be involved in the angiogenesis of lung cancer, as the most frequently changed pathway. Another interesting result found is that all significant pathway pairs had a lower interaction score in lung cancer than in the normal control group. This might indicate that the variability in form of exploited parallel pathways is in cancer lower than in normal cells.

### Gene regulatory networks

Finally, we would like to mention that also gene regulatory network inference methods have also been used in this context. More precisely, several attempts have been made to identify disease networks [110,111] that corresponds to particular pathways. For instance, in [110] the C3NET inference method [116,117] has been used to infer pathway specific networks for prostate cancer. A structural comparison between the pathway-specific networks, similar to [106] which is based on testing the hypothesis in Eqn. 45, allowed to identify growth and cell cycle related pathways.

On a side note, we would like to add that *Gaussian graphical models* (GGM), also known as *Markov random fields*[118-120], are also frequently used to infer gene regulatory networks. This model assumes that all variables follow a multivariate normal distribution with a specific structure of the inverse of the covariance matrix, ***Ω*** = ***Σ***^−1^, whereas ***Ω*** is called the precision or concentration matrix. Network inference methods based on GGM make use of the relation,

(55) $$\rho_{\mathrm{ij}|V\setminus \{\mathrm{ij}\}}=-\frac{\omega_{\mathrm{ij}}}{\sqrt{\omega_{\mathrm{ii}}\omega_{\mathrm{jj}}}},$$

connecting the partial correlation coefficient of *full-order* (LHS) with the elements of ***Ω***, *ω*_*ij*_ ∈ ***Ω***. The partial correlation is of full-order (with respect to the number of genes) because *V*∖{*ij*} is the set of all genes excluding *i* and *j*, i.e., the largest possible set of genes not considering *i* and *j*.

Several methodological improvements have been suggested to infer gene regulatory networks based on GGM [121-123]. These methods differ in the way the inverse of the covariance matrix, ***Σ***^−1^, is estimated and in the statistical tests employed to assess significance. The reason for these technical variants comes from a variety of problems. For instance, if the number of samples is smaller than the number of genes, which is typically the case for a microarray data set, the sample covariance matrix is not positive definite and, hence, not invertible. This means that Eqn. 55 cannot be exploited. In order to overcome such practical estimation problems, recently, several extensions based on the LASSO (least absolute shrinkage and selection operator) have been suggested [124-128].

## Importance of multiple hypotheses testing and sample size: An example for differentially expressed genes

**Figure 5:**
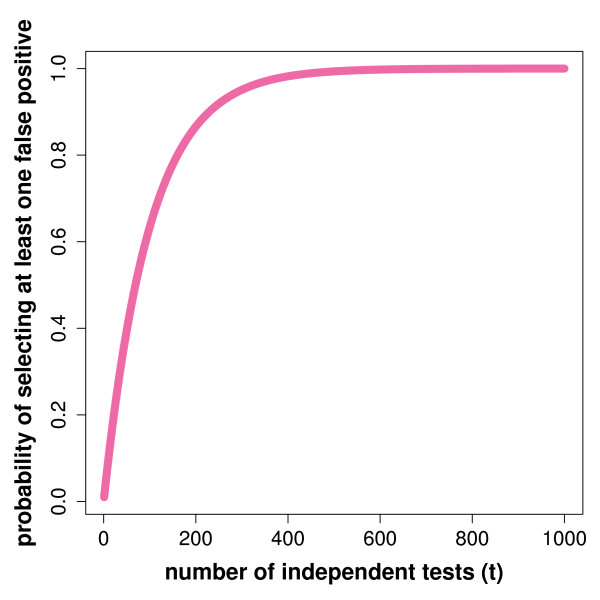
**Dependence of *Pr*** **(*V* ≥ 1|** ***α*  =  0.01, *t*) on the number *t *** **of tested hypotheses.**

Typical microarray experiments measure the concentration of thousands of mRNAs simultaneously. For this reason, usually, one does not just test one statistical hypotheses but dozens, hundreds or even thousands. This makes it mandatory to control the overall error rate for all the tests, because the probability to make *at least* one error, *Pr*(*V* ≥ 1|*α*,*t*) = (1 − (1 − *α*)^*t*^), for a test with a false positive rate of *α*, increases rapidly with the number of tests *t*, as can be seen in Figure 5. Here, *V * corresponds to the number of false positives. From this one can see that even for a moderate number of tested hypotheses, e.g., 300, this probability is already almost 100%. Hence, each of the three principle types of hypotheses tests discussed in the previous sections are severely effected by this problem.

Classically, a Bonferroni correction is used controlling the Family wise Error Rate (FWER) [129,130],

(56) FWER(α)=P(V≥1).

Unfortunately, this method is often too stringent, which may give no significant results at all. For this reason, alternative error measures and control procedures have been introduced. A recent, very popular measure is the false discovery rate (FDR) [131],

(57) $$\mathrm{FDR}=\left\{\begin{array}{cc} E[V/R] & \text{if}\;R>0\\ 0\; & \text{if}\;R=0. \end{array},\;\right\}$$

controlled by a procedure introduced by Benjamini & Hochberg (BH) [131]. Subsequently, various related error measures have been proposed like pFDR [132], local FDR [133,134] and a variety of other control procedures [129,135]. Also extensions have been suggested [136] that allow the control of an error measure in cases where the underlying tests are not independent from each other. This is particularly important for microarray data that contain a none neglectable correlation structure among the genes.

**Figure 6:**
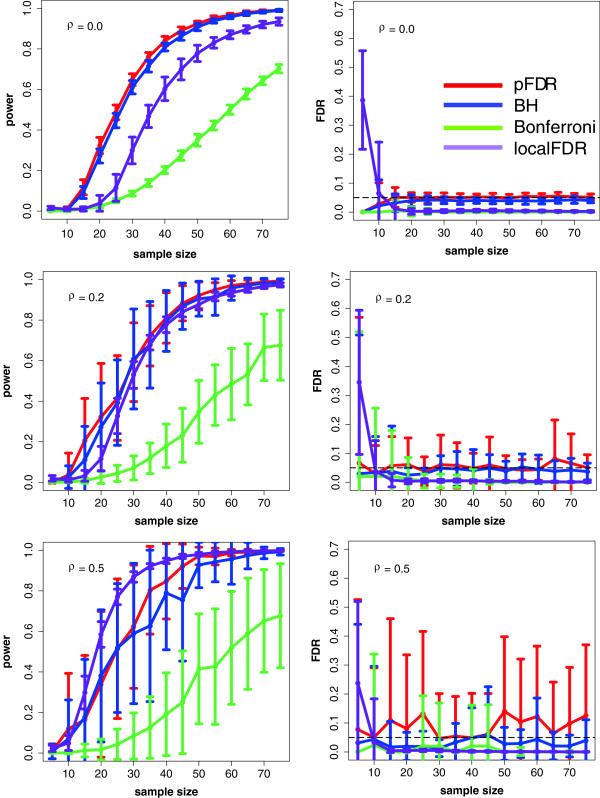
**Power and false discovery rate in dependence on the sample size and the correlation in the data.** We compare the methods: pFDR (red) [138], BH (blue) [131], Bonferroni (green) and localFDR (purple) [134,137].

In order to demonstrate the importance and the influence of different error measures and control procedures, we provide a numerical example, shown in Figure 6. Here, ‘BH’ means the Benjamini & Hochberg procedure to control the FDR [131], ‘pFDR’ indicates the positive false positive rate controlled with a method introduced in [132], ‘Bonferroni’ corresponds to the FWER controlled with a Bonferroni correction and ‘localFDR’ corresponds to the local FDR [134,137]. The local FDR is defined as,

(58) localFDR=Prob(null|test statistic).

that means the local FDR is the probability that the null model is true conditioned on the observed test statistic. The data we used for this analysis correspond to simulated gene expression data sampled from a normal distribution with different mean values for the two conditions. More precisely, we simulate 2000 genes of which 400 are differentially expressed (true positives). Further, we study three different (constant) correlation structures with *ρ* = {0.0,0.2,0.5}. The results shown in Figure 6 are for each sample size averaged over 50 independent runs.

As one can see, ‘BH’ and ‘pFDR’ give more significant results and, hence, have a higher power than the Bonferroni correction and the local FDR when there is either no or only a moderate correlation among the genes. However, it is important to note that the utility of these methods depends on the characteristics of the data. For example, if the average correlation in the data is *ρ* = 0.0, then ‘pFDR’ tends to perform best (see Figure 6A). However, when the average correlation in the data increases (*ρ* = 0.5) then the ‘localFDR’ [134,137] becomes preferable. We want to note, for a sample size of 5, the power of the methods is usually very low because only a couple of genes test significant. In addition, a large fraction of these can be false positives. This seems to be especially for the local FDR method a problem.

## Recommendations

In general, there is a trade-off between a high power of a statistical method on one side, which requires a large number of samples, and low experimental costs on the other. For the identification of differentially expressed genes the results in Figure 6 provide some guidelines. Even for the most favorable condition (for *ρ* = 0.0) a study will usually be underpowered for ≤ 20 samples, however, on the other hand, even for 10 samples the Type I error will be well-controlled.

For gene set and pathway-based methods such recommendation are more delicate. In [105] two self-contained (*sum of t-square* and Hotelling’s *T*^2^[84,95]) and one competitive test (GSEA [27]) have been analyzed. As a results, it is suggested not to apply a method unconditionally to all pathways in a given data set, but to *filter* them in order to eliminate conditions for which a method is more likely to cause problems. This can be seen as a reflection of the heterogeneity of cancer, as discussed above in the section ‘Gene expression data and cancer’.

In [105] it has been suggested to filter pathways according to the following criteria: Hotelling’s *T*^2^ should only be applied to pathways with less than 35 genes and a sample size larger than 30. The *sum of t-square* test should only be used for pathways with *DC* > 10*%* (*DC* is detection call; the percentage of differentially expressed genes in a pathway) and a sample size of 25 or larger. GSEA should only be used for pathways with *DC* > 10*%* and a sample size larger than 25. That means for the *sum of t-square* test and GSEA, at least 10% of the genes in a pathway should be differentially expressed for the method to work. However, this is not independent of the correlation structure of the data. In general, in the presence of high correlations a larger number of differentially expressed genes is beneficial for these methods.

It is important to emphasize that these sample sizes are different to the minimal sample sizes necessary in order to avoid in addition that a study is underpowered. For the minimal sample sizes [105] predict a sample size of 59 for Hotelling’s *T*^2^ and 57 for the *sum of t-square* test and 83 for GSEA. Further, in [95] it was found that using the N-statistic with 40 samples (or more) leads to a good control of the Type I error and a satisfactory power for a variety of differing conditions, including different correlations of the data and DC values in the pathways. Further studies reviewing related methods can found in [61,62,65,139,140].

We would like to emphasize that the above recommendations are data dependent. That means it is not possible to judge solely based on the number of samples which method to use. Instead, one needs to estimate characteristics from a particular data set in order to select an appropriate test. This implies, e.g., to estimate the correlation structure and the detection call. In the context of cancer there is an additional problem that needs to be considered. It is known that a tumor is a heterogeneous collection of cells rather than a homogeneous one [26,141]. This translates into the heterogeneity of gene expression data [142] making it even more dangerous to provide general recommendations without considering a particular data set.

On a general note, we would like to highlight that whenever a given data set allows to (I) identify changes in single genes, (II) identify changes in gene sets or pathways, and (III) identify changes in the correlation structure in pathways, then methods from each of the three categories (IIII) should be applied and there is no need to focus on just one of these. The reason for this is that despite the fact that gene set or pathway methods have more explanatory power than methods to identify changes in single genes [64,95] it does not mean that there are no conditions for which single gene methods reveal interesting biological information that may not be obtained by the other types of methods. For instance, the differential expression of a single gene based on changes in the mean (rather than the variance) may be an indicator for the presence of a single signaling chain rather than of many parallel pathways. Hence, this could provide information about the presence of a Mendelian trait or a complex trait that contains a strong monogenetic component. It appears that for such conditions single gene methods have an advantage over gene set or pathway methods, although, the latter methods may be adaptable to such question as well. However, this may require additional effort. In summary, we recommend to use all different approaches (IIII) side-by side, whenever this is permitted by the data, to interrogate the data in the broadest way, because this translates into a diverse set of different biological questions.

Our recommendation complements a common line of thought asking for the combination of different types of data. Although it is certainly true that combining different types of high-throughput data, e.g., from DNA microarray and ChIP-chip experiments, is in general more informative, it is also more time and cost intensive to generate such data combinations. For this reason, frequently, only gene expression data are available. Hence, our review provides a survey of method to get the most out of expression data sets.

Finally, we would like to emphasize that all methods require an appropriate filtering and normalization of the data in order to obtain robust and statistically sensible results [143,144].

## Conclusions and discussion

In the post genomic era, biology transitioned from a ‘gene-centric’ to a systems-focused field. This change is also reflected in the transition from methods to identify ‘differentially expressed single genes’ to approaches for finding ‘differentially changed pathways’. Such a transition is natural, because a systems view is required to understand the complex biological functions inside a cell that are responsible for the observable phenotypic outcomes [9,11,145].

As recent findings in cancer research demonstrate, cancer is a heterogeneous disorder, even within a particular cancer type. For example, breast cancer is currently subcategorized into four major tumor subtypes [146]: basal-like, HER2-enriched, luminal (which can be further reduced) and normal-like tumors. Considering the fact that these results have been achieved by using high-throughput data one can expect further refinements when data from different hight-throughput technologies become available and being combined with each other. For this reason, it appears sensible to assume such a heterogeneity not only on the global, phenotype level, but also within the cells, on the pathway-level. This implies that a pathway-based filtering, as suggested in [105], is necessary to apply a method only selectively, and not unconditional, to cancer pathways.

Regarding potential future directions, we expect to see an increase in methods that target changes in the correlation structure in pathways for three reasons. First, genes and their products do interact with each other. This implies that there exists a correlation structure among these entities that represents, potentially, useful biological information that may be missed by co-expression based methods [106]. Second, the costs to generate high-throughput data are declining, which makes it easier for the experimenter to generate a sufficiently large number of samples that enables such an analysis. This is an important point, since the required sample sizes for a pathway analysis is considerably larger than for single gene analyses. Third, biologically, the hallmarks of cancer point to a few pathways as pivotal elements in the molecular elucidation of carcinogenesis, e.g., Wnt/Notch signaling, Hedgehog signaling or DNA damage control [147-149]. Hence, semantically, pathway studies enable the systematic connection of oncogenes, tumor-suppressor genes and stability genes [150] to provide fundamental insights into causal mechanisms underlying cancer. Unfortunately, the temporary literature especially of methodological papers discuss their results rarely in the framework of the *hallmark pathways*. For this reason, we suggest that future studies aim for a conceptual discussion of their results within this enlightening framework. Not because it provides the final answers to understand cancer [151], but due to the fact that it enables a systematic approach to the emperor of all maladies [152].

## Reviewers’ comments

First of all, we would like to thank all referees for their fruitful suggestions and comments. In the following, we kept our answers to the raised issues short but included our responses in the main text.

### Referee 1: Dr. Arcady Mushegian

The manuscript by Emmert-Streib and colleagues is a review of statistical methods for analysis of gene expression data, but it is also much more than that. It is relatively rare for the statisticians to review all classes of such methods and to give an eminently logical classification not only of the techniques on which the methods are based, but also of the kinds of questions that are asked when applying these methods. This, certainly, is a strength of the work and the reason why it should appeal to the biologists that would like to have a deeper insight into which methods are appropriate to which task at hand.

I have, however, several comments that rank somewhere between suggestions and concerns. Most importantly, the authors propose to distinguish three groups of methods: those that identify changes in single genes, those that identify changes in gene sets or pathways, and those that identify changes in the correlation structure in pathways. (By the way, in the Abstract and elsewhere, the description of the groups is almost the same as above, but “changes” are substituted by “differential changes” - is it not a tautology, in particular when there are only two samples?). Then, in discussing the first two classes of methods, the authors almost in every case give a clear formulation of the question that is being asked of the data, in the form of the statistical hypothesis about the data that is being tested. This is an excellent way of explaining things. Unfortunately, it is not consistently applied: even among these classes of methods, the hypotheses are not mentioned, and then, upon discussing the differential-correlation methods (pp. 15-18), the hypotheses are not explicated at all, except for the IPS method. I think this need to be changed, and the null hypotheses need to be stated for all methods for which this is possible; and if the framework is such that no explicit null hypothesis exist, this needs to be discussed, and the applicable intuitive formulation be given.

#### Reply

We appreciate this suggestion and added to all methods the definition of their null hypothesis. In addition, we extended the discussion in section ‘Formulating biological hypotheses’ explaining why it can be difficult to find a biological interpretation for a null hypothesis and we offer some explanation for this.

#### Question

My other concern is abut Figures 3 and 4. The authors never state what the data points there represent. They must be expression values for two genes, but how are these data collected - are they technical replicates? biological replicates? some kind of ordered series? unordered series such as for example different drug treatments? Does it matter what of the above they are?

#### Reply

We added an explanation of the data, which are simulated data to visualize the principle idea underlying some methods, to the corresponding methods.

#### Question

The third shortcoming of the paper is that there is a significant disconnect between well-covered methodology and the stated goal of discussing the application to cancer biology. In fact, the short discussion about cancer hallmarks is an excellent introduction that points out the way in which analysis of gene expression can lead to the understanding of changes in expression of particular (“hallmark”) pathways. This theme, however, is not followed through. Though occasionally we read that such and such method was applied to analysis of a particular type of cancer, there is never any discussion of what was found in gene expression data that allowed an insight into cancer biology. What happens to the hallmark pathways at the level of gene expression programs? Which methods have been used to support (or maybe question?) which aspects of the hallmark hypothesis? Which pathways were predicted or shown to be differentially regulated at the transcription or mRNA concentration level?

#### Reply

We agree with the reviewer that ‘Which methods have been used to support (or maybe question?) which aspects of the hallmark hypothesis? ’ is an important questions. Unfortunately, the methodology oriented literature does rarely touch this topic in a clear manner. That means in order to extend the paper in this direction we could not survey these issues but would need to establish such results. Instead, the concern in our paper is to propagate such an approach in the context of the presented methods. A discussion has been added to ‘Discussion and conclusions’.

#### Question

Finally, there is the question of, if you will, general biology of transcriptional response. It stands to reason, and indeed has been occasionally shown, that in order for a pathway to be regulated, it may not be necessary to regulate all its components at the same (in this case, mRNA concentration) level. One may find that the gene product amounts are regulated at different levels, or maybe even only one or a few, e.g., rate-limiting, components are regulated at all. This would argue that single gene-based methods may in these cases provide a better clue to the process than pathway-based or gene set enrichment-based methods. It would be interesting to know whether this has been observed in the cancer datasets. A related question is about the rules of thumb in pathway analysis: for example, if a typical pathway (network module?) has a size of N genes, what is the number of genes in this pathway *m* < *N* that would still register as an enrichment in some of the tests that the authors discuss?

#### Reply

This is an important point. We included a discussion of this to section ‘Recommendations’. We added also a discussion of the danger of general suggestions and motivate this by known characteristics of gene expression data from cancer. The problem is twofold. First, each method has its own characteristics under what conditions it works best. Second, data sets from cancer are very heterogeneous so that two data sets containing about the same number of samples can exhibit a very different correlation structure and expression patterns. This holds potentially also for different grades of one cancer type.

Regarding the first question, it appears to us that this is related to the presence or absence of parallel pathways conveying a molecular signal. If for example no parallel pathways exist the detection of differentially expressed genes can provide a robust way to detect functional changes. On the other hand, if there are many alternatives this may not be the case and gene set methods appear to be better suited for such a situation. In general, this kind of cross-method comparisons are not well studied and we are not aware that this has been systematically addressed for cancer or other data sets. One reason for this is that until recently, most data sets contained less than 20 samples per condition, which usually does not permit a robust analysis of gene set or pathway methods and once larger data sets became available the detection of differentially expressed genes was neglected, potentially, due to the erroneous assumption that differentially expressed gene set methods include the former tests.

In order to emphasize that it is desirable to apply methods from all three different levels simultaneously ((I) identify changes in single genes, (II) identify changes in gene sets or pathways, and (III) identify changes in the correlation structure in pathways) whenever a given data sets allows this, rather than to focus on just one of these levels, we added a discussion to section ‘Recommendations’.

Thank you for your suggestions and comments.

### Referee 2: Dr. Byung-Soo Kim

General comments This is a well organized review of recent statistical methods of analyzing microarray experiment data sets, particularly on cancers, from single gene analysis to identifying differential changes in pathway, and finally to comparing a given pathway under two different conditions. However, I would like to indicate following four points for the possible improvement. (1) Gaussian graphical model: From the methodological point of view, it is desired to include the sparse Gaussian graphical model (GGM) approach for estimating the gene network under the multivariate normal assumption from a microarray data set. For the recent development of GGM approach one can include glasso (Friedman, Hastie and Tibshirani, 2008; Witten, Friedman and Simon, 2011) [125,127], SCAD penalty of Fan, Feng and Wu (2009) [124], adaptive lasso of Zou (2006) [128] and Kiiveri (2012) [126], among others. (2) Effect of inter-gene correlations on the single gene analysis. A series of Efron’s recent work (Efron 2007a, 2007b) [134,137] discussed in detail on how inter-gene correlations could affect the detection of differentially expressed (DE) genes in a single gene analysis? By including Efron’s recent work and his R package “locfdr” authors can show how FDR can be used in the real data analysis in their Section on “Importance of multiple hypotheses testing and sample size: An example for differentially expressed genes”. (3) Some of the reviews are misleading. These are the few examples. (i) The sentence, at the middle of page 12, “However, in order to use a two-sample t-test with equal size of the two samples it is assumed that the mean fold change f’ and its standard deviation *σ*_*f*_ would be the same for a randomly selected background set consisting of only m genes, see Eqn. 10”. Actually ([99], Luo et al., 2009) assumes the i.i.d of the fold change of genes to make Eqn 10 have a t distribution. Here the key assumption was the independence, which was missing in the aforementioned sentence. (ii) p. 14. Eqn 16. In ([126], Tian et al., 2005) no t-square statistic was employed. (iii) Eqn 24 of p. 18 does not make sense. Authors of ([20], Cho et al., 2009) didn’t make it clear in their equation (3) what Renyi entropy was when the underlying random variables were continuous. (iv) I would suggest authors to allocate more space on the work of ([90], Massa et al, 2010) which was methodologically sound and deserve more coverage than just the IPS algorithm. (4) Inconsistency of notations. In page 11 authors defined p and m to be sizes of the background genes and a target gene set, respectively. However, in line 2 of page 15 “p genes” (which should have been m genes according to page 11 definition) was incorrectly labeled. This inconsistency was repeated in N-statistic section of p.15, and also in Eqn 16 in p. 14 and Eqn 22 of p. 17. The “p-dimensional..” should be “m-dimensional..” at the bottom two lines of p. 14.

#### Minor Comments

1. p.2 “Gene expression data from next generation sequencing (RNA-seq)”. This is an important issue. There is no direct relevance, however, with statistical methods reviewed in this paper.

2. p.4. For detecting differential correlation and differential variance, it would be better to explain why these approaches were taken. For example, in ([54], Ho et al., 2008) it was clearly indicated that changes in expression variability were associated with changes in coexpression pattern, which implied that DV was a signal rather than a noise.

3. Legend of Figure 2. “The data is..” should be “The data are.. ”.

4. p.7. There is no reference of Figures A, B in the main text. Also indicate in the legend of Figure 3 what *Σ* is in Figure 3E.

5. p.8. In the legend of Figure 4 what the symbols in the outer-panel represent? What do the lines represent? It is better to use different notation (A, B) to avoid confusion in the main text of the second paragraph of p. 9.

6. p.9 What is “alpha” in Equation (4)?

7. p.9 line -9. You may include two specific patterns of dependence of two genes, namely, type A dependence of Klevanov, Jordan and Yakovlev (2006), and hidden regulator dependence of Lim, Kim and Kim (2011).

8. p.15 line -13. “euclidean Kernel” should be “Euclidean kernel” (9)

9. p.15 line -10. “a either” should be “either”.

10. p.15 line -8. Author may want to include Tsai and Chen (2009) for another reference of Hotelling’s T-square statistic.

11. p.17. line 15, What are “A” and “B”?

12. p. 18 line 2. Better to include Lauritzen (1996) as a reference of IPS algorithm.

13. p. 22. It would be more beneficial for the read to move the last paragraph of p. 22 (extended to p. 23) to Introduction section.

References Efron B. (2007a). Correlation and large-scale simultaneous signifance testing, J. Amer. Statist. Assoc. 102:93-103. Efron B. (2007b). Size, power and false discovery rates, Annals of Statist. 35:1351-1377. Fan J, Feng Y, Wu Y. (2009). Network exploration via the adaptive lasso and SCAD penalties. Ann. Statist. 3:521-541. Friedman J, Hastie T, Tibshirani R. (2008). Sparse inverse covariance estimation with the graphical lasso. Biostatistics 9:432-441. Lauritzen SL. (1996), Graphical models, Oxford: Clarendon Press. Lim J, Kim J, Kim BS (2011). An alternative model of type A dependence in a gene set of correlated genews, Statist. Appl. Genet. Mol. Biol. Vol. 9, Article 12. Kiiver H, de Hoog F. (2012). Fitting very large Gaussian graphical models. Comp. Statist. Data Anal. 56:2626-2636. Klebanov L, Jordan C, Yakovlev A. (2006). A new type of stochastic dependence revealed in gene expression data, Statist. Appl. Genet. Mol. Biol. Vol. 5, Article 7. Tsai C-A, Chen J. (2009). Multivariate analysis of variance test for gene set analysis, Bioinformatics, 25:897-903. Witten DM, Friedman JH, Simon N. (2011). New insights and faster computation for the graphical lasso. J. Comp. Graph. Stat. 20:892-900. Zou H. (2006). The adaptive lasso and its oracle properties. J. Amer. Statist. Assoc. 101:1418-1429.

#### Reply

We revised our text correspondingly and addressed all your suggestions. We would like to point out that the major goal of our review is not a full coverage of statistical details but to provide sufficient information for the reader to acquire a basic understanding of major principles and assumptions that underly the methods. The problem is that if too many detail are presented the paper would turn quickly into a formal description which may not be appreciated by a biology oriented readership.

#### Minor Comments

1. p.12. line 1: What is N?

2. p. 15. line -7 -5: “two i.i.d samples of genes..” is rather confusing. Luo et al. (2005) assumed the i.i.d of the fold change of genes, which was much stronger than just assuming equal mean and variance. It is better to rewrite this sentence to convey the original material.

3. p.17. line -1: “p genes” should be “m genes”.

4. p.22. Eqn. (50): What are p and q? What are *S*_*i*_ and *S*_*j*_?

5. p.37. Reference 30, p. 38. References 40, 59; The journal title should be consistent with Reference 27 or vise versa.

6. p.40. References 90, 108: Location of the publisher is missing.

7. p.40. References 93,94; The journal title should be consistent with Reference 119.

8. p.40. Reference 109: Author was duplicated at the end. The location and the publisher were missing.

9. p.41. Reference 117. The article title is missing.

10. p.41. Reference 118: The location of the publisher is missing.

11. p.41. Reference 133. The journal title should be consistent with Reference 27.

#### Reply

All comments have been addressed and we revised the main text correspondingly.

Thank you for your suggestions and comments.

### Referee 3: Dr. Joel Bader

This manuscript reviews methods for analyzing gene expression data with tests of individual genes, gene pairs, gene sets, and networks. The manuscript is strong in covering many methods. It would be more helpful if the authors also provided a point of view or evaluation of methods. Can anything be said about the relative power of different approaches, or which have proven to be more useful in practice? What about the tradeoff between robustness, power, and speed for realistic data? Most of the discussion of method choice is generally about sample size requirements for all methods rather than method choice given sample size. The two parts of the manuscript, gene expression and cancer, don’t really mesh. Most of the methods review is not cancer specific. Possibly of greater relevance to cancer are methods that combine different types of data.

The manuscript is generally well written and easy to understand, with ample references to the original work and to previous reviews.

Minor corrections

1. p. 1 ‘one gene, −> should be ‘ for open-quote in latex, here and elsewhere

2. p. 2 differnt microarray −> spelling

3. p. 2 comprises, e.g., mRNAs −> ‘e.g.’ doesn’t sound right here. How about providing a full list: mRNA, tRNA, rRNA, and short regulatory RNAs

4. p. 2 ‘In the third step the reads are mapped to known exon sequences of genes.’ Are there also de novo assembly methods that don’t require a template? ‘allows to overcome’ −> overcomes

5. p. 3 allows to measure −> measures. Can also mention other advantages: splice variants, sequence polymorphisms, no need to design and build a custom chip

6. ‘correspond to: self sufficiency’ −> no colon between preposition and noun phrase. Can the hallmarks be parallel, all start with noun or verb?

7. p. 9 Eq. 4. How is alpha calculated?

8. Eq. 5 need i = 1 underneath the summation

9. p. 14. Eq. 16 Under the null, it seems that *Σ*_*t*,2_should approach $1/\sqrt{(}p)$ rather than 0.

10. Eq. 16 How is the significance of SAM-GS calculated?

11. p. 18 Eq. 24 and text after, use log in math mode rather than log.

#### Reply

All comments have been addressed and we revised the main text correspondingly.

Thank you for your suggestions and comments.

## Competing interests

The authors declare that they have no competing interests.

## Author’s contributions

ST simulated and visualized the data. ST, RDMS and FES analyzed and interpreted the results. ST, RDMS and FES wrote the paper. FES conceived the paper. All authors read and approved the final manuscript.

## References

[B1] Bock G Goode J Novartis Foundation Symposium 1998 John Wiley & Sons

[B2] Van Regenmortel M Reductionism and complexity in molecular biology EMBO reports 2004 5 9 1016 1020 15520799 10.1038/sj.embor.7400284 PMC1299179

[B3] Mazzocchi F Complexity in biology EMBO Rep 2008 9 10 14 18174892 10.1038/sj.embor.7401147 PMC2246621

[B4] von Bertalanffy L An outline of general systems theory Br J Philosophy Sci 1950 1 2 134 165

[B5] Beadle GW Tatum EL Genetic control of biochemical reactions in neurospora Proc Natl Acad Sci USA 1941 27 11 499 506 16588492 10.1073/pnas.27.11.499 PMC1078370

[B6] Hanahan D Weinberg RA The hallmarks of cancer Cell 2000 100 57 70 10647931 10.1016/s0092-8674(00)81683-9

[B7] Noble D Genes and causation Phil Trans R Soc A 2008 366 3001 03015 18559318 10.1098/rsta.2008.0086

[B8] Kitano H Systems biology: a brief overview Science 2002 295 5560 1662 1664 11872829 10.1126/science.1069492

[B9] Han JDJ Understanding biological functions through molecular networks Cell Res 2008 18 2 224 237 18227860 10.1038/cr.2008.16

[B10] MacDougall-Shackleton SA The levels of analysis revisited Phil Trans R Soc B: Biol Sci 2011 366 1574 2076 2085 10.1098/rstb.2010.0363 PMC3130367 21690126

[B11] Barabasi AL Oltvai ZN Network biology: understanding the cell’s functional organization Nat Rev 2004 5 101 113 10.1038/nrg1272 14735121

[B12] Brazhnik P de la Fuente A Mendes P Gene networks: how to put the function in genomics Trends Biotechnol 2002 20 11 467 472 12413821 10.1016/s0167-7799(02)02053-x

[B13] Emmert-Streib F Glazko G Network biology: a direct approach to study biological function Wiley Interdiscip Rev Syst Biol Med 2011 3 4 379 391 21197659 10.1002/wsbm.134

[B14] Davidson E Levin M Gene regulatory networks Proc Natl Acad Sci USA 2005 102 14 4935 15809445 10.1073/pnas.0502024102 PMC556010

[B15] de Matos Simoes R Tripathi S Emmert-Streib F Organizational structure of the peripheral gene regulatory network in B-cell lymphoma BMC Syst Biol 2012 6 38 22583750 10.1186/1752-0509-6-38 PMC3476434

[B16] Jones S Thornton JM Principles of protein-protein interactions Proc Nat Acad Sci 1996 93 13 20 8552589 10.1073/pnas.93.1.13 PMC40170

[B17] Maslov S Sneppen K Specificity and stability in topology of protein networks Science 2002 296 5569 910 913 11988575 10.1126/science.1065103

[B18] Jeong H Tombor B Albert R Olivai Z Barabasi A The large-scale organization of metabolic networks Nature 2000 407 651 654 11034217 10.1038/35036627

[B19] Babu MM Luscombe NM Aravind L Gerstein M Teichmann SA Structure and evolution of transcriptional regulatory networks Curr Opin Struct Biol 2004 14 283 291 15193307 10.1016/j.sbi.2004.05.004

[B20] Lee TI Transcriptional regulatory networks in saccharomyces cerevisiae Science 2002 298 5594 799 804 12399584 10.1126/science.1075090

[B21] Allison DB Microarray data analysis: from disarray to consolidation and consensus Nat Rev Genet 2006 7 55 65 16369572 10.1038/nrg1749

[B22] Dehmer M Emmert-Streib F Graber A Salvador A(Eds) Applied Statistics for Network Biology: Methods for Systems Biology 2011 Weinheim: Wiley-Blackwell

[B23] Quackenbush J Computational analysis of microarray data Nat Rev Genet 2001 2 6 418 427 11389458 10.1038/35076576

[B24] Metzker ML Sequencing technologies - the next generation (With NOTES) Nat Rev Genet 2010 11 31 46 19997069 10.1038/nrg2626

[B25] Wang Z Gerstein M Snyder M RNA-Seq: a revolutionary tool for transcriptomics Nat Rev Genet 2009 10 57 63 19015660 10.1038/nrg2484 PMC2949280

[B26] Hanahan D Weinberg RA Hallmarks of cancer: the next generation Cell 2011 144 5 646 674 21376230 10.1016/j.cell.2011.02.013

[B27] Subramanian A Tamayo P Mootha V Mukherjee S Ebert B Gillette M Paulovich A Pomeroy S Golub T Lander E Mesirov J Gene set enrichment analysis: a knowledge-based approach for interpreting genome-wide expression profiles Proc Natl Acad Sci USA 2005 102 43 15545 50 16199517 10.1073/pnas.0506580102 PMC1239896

[B28] Chuang HY Lee E Liu YT Ideker T Network-based classification of breast cancer metastasis Mol Syst Biol 2007 3 140 17940530 10.1038/msb4100180 PMC2063581

[B29] Compagno M Lim WK Grunn A Nandula SV Brahmachary M Shen Q Bertoni F Ponzoni M Scandurra M Califano A Mutations of multiple genes cause deregulation of NF-kappaB in diffuse large B-cell lymphoma Nature 2009 459 7247 717 721 19412164 10.1038/nature07968 PMC2973325

[B30] Horvath S Zhang B Carlson M Lu KV Zhu S Felciano RM Laurance MF Zhao W Qi S Chen Z Analysis of oncogenic signaling networks in glioblastoma identifies ASPM as a molecular target Proc Natl Acad Sci USA 2006 103 46 17402 17407 17090670 10.1073/pnas.0608396103 PMC1635024

[B31] Krivtsov AV Twomey D Feng Z Stubbs MC Wang Y Faber J Levine JE Wang J Hahn WC Gilliland DG Transformation from committed progenitor to leukaemia stem cell initiated by MLL-AF9 Nature 2006 442 7104 818 822 16862118 10.1038/nature04980

[B32] Oskarsson T Acharyya S Zhang XHF Vanharanta S Tavazoie SF Morris PG Downey RJ Manova-Todorova K Brogi E Massague J Breast cancer cells produce tenascin C as a metastatic niche component to colonize the lungs Nat Med 2011 17 7 867 874 21706029 10.1038/nm.2379 PMC4020577

[B33] Mavrakis KJ Wolfe AL Oricchio E Palomero T De Keersmaecker K McJunkin K Zuber J James T Khan AA Leslie CS Genome-wide RNA-mediated interference screen identifies miR-19 targets in Notch-induced T-cell acute lymphoblastic leukaemia Nat Cell Biol 2010 12 4 372 379 20190740 10.1038/ncb2037 PMC2989719

[B34] Nam S Park T Pathway-based evaluation in early onset colorectal cancer suggests focal adhesion and immunosuppression along with Epithelial-Mesenchymal transition PLoS ONE 2012 7 4 e31685 22496728 10.1371/journal.pone.0031685 PMC3322137

[B35] Guedj M Marisa L De Reynies A Orsetti B Schiappa R Bibeau F Macgrogan G Lerebours F Finetti P Longy M A refined molecular taxonomy of breast cancer Oncogene 2011 31 July 2011 1196 1206 21785460 10.1038/onc.2011.301 PMC3307061

[B36] Lehmann BD Bauer JA Chen X Sanders ME Chakravarthy AB Shyr Y Pietenpol JA Identification of human triple-negative breast cancer subtypes and preclinical models for selection of targeted therapies J Clin Invest 2011 121 7 2750 2767 21633166 10.1172/JCI45014 PMC3127435

[B37] Fabbri G Rasi S Rossi D Trifonov V Khiabanian H Ma J Grunn A Fangazio M Capello D Monti S Analysis of the chronic lymphocytic leukemia coding genome: role of NOTCH1 mutational activation J Exp Med 2011 208 7 1389 1401 21670202 10.1084/jem.20110921 PMC3135373

[B38] Ooi CH Ivanova T Wu J Lee M Tan IB Tao J Ward L Koo JH Gopalakrishnan V Zhu Y Cheng LL Lee J Rha SY Chung HC Ganesan K So J Soo KC Lim D Chan WH Wong WK Bowtell D Yeoh KG Grabsch H Boussioutas A Tan P Oncogenic pathway combinations predict clinical prognosis in gastric cancer PLoS Genet 2009 5 10 e1000676 19798449 10.1371/journal.pgen.1000676 PMC2748685

[B39] Setlur SR Royce TE Sboner A Mosquera JM Demichelis F Hofer MD Mertz KD Gerstein M Rubin MA Integrative microarray analysis of pathways dysregulated in metastatic prostate cancer Cancer Res 2007 67 21 10296 10303 17974971 10.1158/0008-5472.CAN-07-2173

[B40] Nucera C Porrello A Antonello ZA Mekel M Nehs MA Giordano TJ Gerald D Benjamin LE Priolo C Puxeddu E B-Raf(V600E) and thrombospondin-1 promote thyroid cancer progression Proc Natl Acad Sci USA 2010 107 23 10649 10654 20498063 10.1073/pnas.1004934107 PMC2890809

[B41] Shah MA Khanin R Tang L Janjigian YY Klimstra DS Gerdes H Kelsen DP Molecular classification of gastric cancer: a new paradigm Clin Cancer Res 2011 17 9 2693 2701 21430069 10.1158/1078-0432.CCR-10-2203 PMC3100216

[B42] Perroud B Lee J Valkova N Dhirapong A Lin PY Fiehn O Kultz D Weiss R Pathway analysis of kidney cancer using proteomics and metabolic profiling Mol Cancer 2006 5 64 17123452 10.1186/1476-4598-5-64 PMC1665458

[B43] Trewavas A A Brief History of Systems Biology: “Every object that biology studies is a system of systems.” Francois Jacob (1974) Plant Cell 2006 18 10 2420 2430 17088606 10.1105/tpc.106.042267 PMC1626627

[B44] Emmert-Streib F Dehmer M Networks for systems biology: conceptual connection of data and function IET Syst Biol 2011 5 3 185 21639592 10.1049/iet-syb.2010.0025

[B45] Macneil LT Walhout AJM Gene regulatory networks and the role of robustness and stochasticity in the control of gene expression Genome Res 2011 21 5 645 57 21324878 10.1101/gr.097378.109 PMC3083081

[B46] Lehman E Testing Statistical Hypotheses 2005 New York: Springer

[B47] DasGupta A Probability for Statistics and Machine Learning 2011 New York: Springer

[B48] Chen Y Dougherty ER Bittner ML Ratio-based decisions and the quantitative analysis of cDNA microarray smages J Biomed Optics 1997 2 4 364 374 10.1117/12.281504 23014960

[B49] Zhang L Zhou W Velculescu VE Kern SE Hruban RH Hamilton SR Vogelstein B Kinzler KW Gene expression profiles in normal and cancer cells Science 1997 276 5316 1268 1272 9157888 10.1126/science.276.5316.1268

[B50] Tusher V Tibshirani R Chu G Significance analysis of microarrays applied to the ionizing radiation response Proc Natl Acad Sci USA 2001 98 18 5116 5121 11309499 10.1073/pnas.091062498 PMC33173

[B51] Chu G Narasimhan B Tibshirani R Tusher V Significance analysis of microarrays (SAM) software Nature 2002 5 436 442

[B52] Smyth GK Gentleman R, Carey V, Dudoit S, Irizarry R, Huber W Limma: linear models for microarray data Bioinformatics and Computational Biology Solutions using R and Bioconductor 2005 New York: Springer 397 420

[B53] Efron B Tibshirani R JD S Tusher V Empirical Bayes analysis of a microarray experiment J Am Stat Assoc 2001 96 456 1151 1160

[B54] Ho JWK Stefani M Dos Remedios CG Charleston MA Differential variability analysis of gene expression and its application to human diseases Bioinformatics 2008 24 13 i390 i398 18586739 10.1093/bioinformatics/btn142 PMC2718620

[B55] Hu R Qiu X Glazko G Klebanov L Yakovlev A Detecting intergene correlation changes in microarray analysis: a new approach to gene selection BMC Bioinformatics 2009 10 20 19146700 10.1186/1471-2105-10-20 PMC2657217

[B56] Dettling M Gabrielson E Parmigiani G Searching for differentially expressed gene combinations Genome Biol 2005 6 10 R88 16207359 10.1186/gb-2005-6-10-r88 PMC1257471

[B57] Lai Y Wu B Chen L Zhao H A statistical method for identifying differential gene-gene co-expression patterns Bioinformatics 2004 20 17 3146 3155 15231528 10.1093/bioinformatics/bth379

[B58] Dawson JA Ye S Kendziorski C R/EBcoexpress: an empirical Bayesian framework for discovering differential co-expression Bioinformatics 2012 28 14 1939 1940 22595207 10.1093/bioinformatics/bts268 PMC3492001

[B59] Li KC Genome-wide coexpression dynamics: theory and application Proc Natl Acad Sci USA 2002 99 16875 16880 12486219 10.1073/pnas.252466999 PMC139237

[B60] Ashburner M Ball CA Blake JA Botstein D Butler H Cherry JM Davis AP Dolinski K Dwight SS Eppig JT Harris MA Hill DP Issel-Tarver L Kasarskis A Lewis S Matese JC Richardson JE Ringwald M Rubin GM Sherlock G Gene ontology: tool for the unification of biology. The gene ontology consortium Nature Genet 2000 25 25 29 10802651 10.1038/75556 PMC3037419

[B61] Ackermann M Strimmer K A general modular framework for gene set enrichment analysis BMC Bioinformatics 2009 10 47 19192285 10.1186/1471-2105-10-47 PMC2661051

[B62] Dinu I Potter JD Mueller T Liu Q Adewale AJ Jhangri GS Einecke G Famulski KS Halloran P Yasui Y Gene-set analysis and reduction Brief Bioinform 2009 10 24 34 18836208 10.1093/bib/bbn042 PMC2638622

[B63] Emmert-Streib F Glazko G Pathway analysis of expression data: deciphering functional building blocks of complex diseases PLoS Comput Biology 2011 7 5 e1002053 10.1371/journal.pcbi.1002053 PMC3102754 21637797

[B64] Khatri P Sirota M Butte A J Ten years of pathway analysis: current approaches and outstanding challenges PLoS Comput Biol 2012 8 2 e1002375 22383865 10.1371/journal.pcbi.1002375 PMC3285573

[B65] Liu Q Dinu I Adewale A Potter J Yasui Y Comparative evaluation of gene-set analysis methods BMC Bioinformatics 2007 8 431 17988400 10.1186/1471-2105-8-431 PMC2238724

[B66] Goeman J Buhlmann P Analyzing gene expression data in terms of gene sets: methodological issues Bioinformatics 2007 23 8 980 7 17303618 10.1093/bioinformatics/btm051

[B67] Huang DW Sherman BT Lempicki RA Bioinformatics enrichment tools: paths toward the comprehensive functional analysis of large gene lists Nucl Acids Res 2009 37 1 13 19033363 10.1093/nar/gkn923 PMC2615629

[B68] Mootha V Lindgren C Eriksson KFea PGC-1alpha-responsive genes involved in oxidative phosphorylation are coordinately downregulated in human diabetes Nature Genet 2003 34 267 273 12808457 10.1038/ng1180

[B69] Efron B Tibshiran R On testing the significance of sets of genes Ann Appl Stat 2007 1 107 129

[B70] Dørum G Snipen L Solheim M Sæbø S Rotation testing in gene set enrichment analysis for small direct comparison experiments Stat App Genet Mol Biol 2009 8 34 10.2202/1544-6115.1418 19645689

[B71] Luo W Friedman M Shedden K Hankenson K Woolf P GAGE: generally applicable gene set enrichment for pathway analysis BMC Bioinformatics 2009 10 161 19473525 10.1186/1471-2105-10-161 PMC2696452

[B72] Kim SY Volsky D PAGE: Parametric Analysis of Gene Set Enrichment BMC Bioinformatics 2005 6 144 15941488 10.1186/1471-2105-6-144 PMC1183189

[B73] Newton M Quintana F den Boon Jea Random-set methods identify distinct aspects of the enrichment signal in gene-set analysis Ann Appl Stat 2007 1 85 106

[B74] Freudenberg JM Sivaganesan S Phatak M Shinde K Medvedovic M Generalized random set framework for functional enrichment analysis using primary genomics datasets Bioinformatics 2011 Jan 1 27 1 70 7 20971985 10.1093/bioinformatics/btq593 PMC3025713

[B75] Rafael IA Chi W Yun Z Terence SP Gene set enrichment analysis made simple Stat Methods Med Res 2009 18 6 565 575 20048385 10.1177/0962280209351908 PMC3134237

[B76] Lange K Numerical Analysis for Statisticians. Statistics and Computing 2010 Springer

[B77] Pyeon D Newton MA Lambert PF den Boon JA Sengupta S Marsit CJ Woodworth CD Connor JP Haugen TH Smith EM Kelsey KT Turek LP Ahlquist P Fundamental differences in cell cycle deregulation in human Papillomavirus-positive and human Papillomavirus-negative head/neck and cervical cancers Cancer Res 2007 67 10 4605 4619 17510386 10.1158/0008-5472.CAN-06-3619 PMC2858285

[B78] Sheskin DJ Handbook of Parametric and Nonparametric Statistical Procedures3rd edition. Boca Raton: RC Press; 2004.

[B79] Tian L Greenberg SA Kong SW Altschuler J Kohane IS Park PJ Discovering statistically significant pathways in expression profiling studies Proc Natl Acad Sci USA 2005 102 38 13544 13549 16174746 10.1073/pnas.0506577102 PMC1200092

[B80] Jiang Z Gentleman R Extensions to gene set enrichment Bioinformatics 2007 23 3 306 313 17127676 10.1093/bioinformatics/btl599

[B81] Dinu I Potter JD Mueller T Liu Q Adewale AJ Jhangri GS Einecke G Famulski KS Halloran P Yasui Y Improving gene set analysis of microarray data by SAM-GS BMC Bioinformatics 2007 8 242 17612399 10.1186/1471-2105-8-242 PMC1931607

[B82] Goeman JJ van de Geer SA de Kort F van Houwelingen HC A global test for groups of genes: testing association with a clinical outcome Bioinformatics 2004 20 93 99 14693814 10.1093/bioinformatics/btg382

[B83] Hummel M Meister R Mansmann U GlobalANCOVA: exploration and assessment of gene group effects Bioinformatics 2008 24 78 85 18024976 10.1093/bioinformatics/btm531

[B84] Lu Y Liu P Xiao P Deng H Hotelling’s T2 multivariate profiling for detecting differential expression in microarrays Bioinformatics 2005 21 14 3105 3113 15905280 10.1093/bioinformatics/bti496

[B85] Kong S Pu W Park P A multivariate approach for integrating genome-wide expression data and biological knowledge Bioinformatics 2006 22 19 2373 2380 16877751 10.1093/bioinformatics/btl401 PMC2813864

[B86] Tsai C Chen J Multivariate analysis of variance test for gene set analysis Bioinformatics 2009 25 7 897 903 19254923 10.1093/bioinformatics/btp098

[B87] Xiong H Non-linear tests for identifying differentially expressed genes or genetic networks Bioinformatics 2006 22 8 919 923 16473873 10.1093/bioinformatics/btl034

[B88] Klebanov L Glazko G Salzman P Yakovlev A Xiao Y A multivariate extension of the gene set enrichment analysis J Bioinform Comput Biol 2007 5 5 1139 1153 17933015 10.1142/s0219720007003041

[B89] Yates P Reimers M RCMAT: a regularized covariance matrix approach to testing gene sets BMC Bioinformatics 2009 10 300 19772589 10.1186/1471-2105-10-300 PMC3087342

[B90] Draghici S Khatri P Tarca AL Amin K Done A Voichita C Georgescu C Romero R A systems biology approach for pathway level analysis Genome Res 2007 17 10 1537 1545 17785539 10.1101/gr.6202607 PMC1987343

[B91] Tarca AL Draghici S Khatri P Hassan SS Mittal P Kim JS Kim CJ Kusanovic JP Romero R A novel signaling pathway impact analysis Bioinformatics 2009 25 75 82 18990722 10.1093/bioinformatics/btn577 PMC2732297

[B92] Thomas R Gohlke JM Stopper GF Parham FM Portier CJ Choosing the right path: enhancement of biologically relevant sets of genes or proteins using pathway structure Genome Biol 2009 10 4 R44 19393085 10.1186/gb-2009-10-4-r44 PMC2688935

[B93] Vaske CJ Benz SC Sanborn JZ Earl D Szeto C Zhu J Haussler D Stuart JM Inference of patient-specific pathway activities from multi-dimensional cancer genomics data using PARADIGM Bioinformatics 2010 26 12 i237—i245 20529912 10.1093/bioinformatics/btq182 PMC2881367

[B94] Massa M Chiogna M Romualdi C Gene set analysis exploiting the topology of a pathway BMC Syst Biol 2010 4 121 20809931 10.1186/1752-0509-4-121 PMC2945950

[B95] Glazko G Emmert-Streib F Unite and conquer: univariate and multivariate approaches for finding differentially expressed gene sets Bioinformatics 2009 25 18 2348 2354 19574285 10.1093/bioinformatics/btp406 PMC2735665

[B96] Ledoit O Wolf M Improved estimation of the covariance matrix of stock returns with an application to portfolio selection J Empir Finance 2003 10 603 621

[B97] Ledoit O Wolf M A well conditioned estimator for largedimensional covariance matrices J Multiv Anal 2004 88 365 411

[B98] Ledoit O Wolf M Honey, I shrunk the sample covariance matrix J Portfolio Manage 2004 30 110 119

[B99] Schäfer J Strimmer K A shrinkage approach to large-scale covariance matrix estimation and implications for functional Genomics Stat Appl Genet Mol Biol 2005 4 32 10.2202/1544-6115.1175 16646851

[B100] Kanehisa M Goto S KEGG: kyoto encyclopia of genes and genomes Nuclei Acids Res 2000 28 27 30 10.1093/nar/28.1.27 PMC102409 10592173

[B101] Lauritzen S Graphical Models 1996 New York: Oxford Science Publications, Clarendon Press

[B102] Matthews L Gopinath G Gillespie M Caudy M Croft D de Bono B Garapati P Hemish J Hermjakob H Jassal B Kanapin A Lewis S Mahajan S May B Schmidt E Vastrik I Wu G Birney E Stein L D’Eustachio P Reactome knowledgebase of human biological pathways and processes Nucleic Acids Res 2009 37 suppl 1 D619—D622 18981052 10.1093/nar/gkn863 PMC2686536

[B103] Klebanov L Jordan C Yakovlev A A new type of stochastic dependence revealed in gene expression data Stat Appl Genet Mol Biol 2006 5 05/11 Article7 16646871 10.2202/1544-6115.1189

[B104] Lim J Kim J Kim B An alternative model of type A dependence in a gene set of correlated genes Stat Appl in Genet Mol Biol 2010 9 Article 12 20196747 10.2202/1544-6115.1525

[B105] Tripathi S Emmert-Streib F Assessment method for a power analysis to identify differentially expressed pathways PLoS ONE 2012 7 5 e37510 22629411 10.1371/journal.pone.0037510 PMC3356338

[B106] Emmert-Streib F The chronic fatigue syndrome: a comparative pathway analysis J Comput Biol 2007 14 7 961 972 17803373 10.1089/cmb.2007.0041

[B107] Choi Y Kendziorski C Statistical methods for gene set co-expression analysis Bioinformatics 2009 25 21 2780 2786 19689953 10.1093/bioinformatics/btp502 PMC2781749

[B108] Cho SB Kim J Kim JH Identifying set-wise differential co-expression in gene expression microarray data BMC Bioinformatics 2009 10 109 19371436 10.1186/1471-2105-10-109 PMC2679020

[B109] Tesson BM Breitling R Jansen RC DiffCoEx: a simple and sensitive method to find differentially coexpressed gene modules BMC Bioinformatics 2010 11 497 20925918 10.1186/1471-2105-11-497 PMC2976757

[B110] Altay G Asim M Markowetz F Neal DE Differential C3NET reveals disease networks of direct physical interactions BMC Bioinformatics 2011 12 296 21777411 10.1186/1471-2105-12-296 PMC3156794

[B111] Watkinson J Wang X Zheng T Anastassiou D Identification of gene interactions associated with disease from gene expression data using synergy networks BMC Syst Biol 2008 2 10 18234101 10.1186/1752-0509-2-10 PMC2258206

[B112] Bunke H What is the distance between graphs? Bull EATCS 1983 20 35 39

[B113] Fuite J Vernon S Broderick G Neuroendocrine and immune network re-modeling in chronic fatigue syndrome: an exploratory analysis Genomics 2008 92 393 399 18775774 10.1016/j.ygeno.2008.08.008

[B114] Wang YC Lan CY Hsieh WP Murillo L Agabian N Chen BS Global screening of potential Candida albicans biofilm-related transcription factors via network comparison BMC Bioinformatics 2010 11 53 20102611 10.1186/1471-2105-11-53 PMC2842261

[B115] Gill R Datta S Datta S A statistical framework for differential network analysis from microarray data BMC Bioinformatics 2010 11 95 20170493 10.1186/1471-2105-11-95 PMC2838870

[B116] Altay G Emmert-Streib F Inferring the conservative causal core of gene regulatory networks BMC Syst Biol 2010 4 132 20920161 10.1186/1752-0509-4-132 PMC2955605

[B117] Altay G Emmert-Streib F Structural influence of gene networks on their inference: analysis of C3NET Biol Direct 2011 6 31 21696592 10.1186/1745-6150-6-31 PMC3136421

[B118] Dempster A Covariance selection Biometrics 1972 28 157 175

[B119] Koller D Friedman N Probabilistic Graphical Models: Principles and Techniques 2009 Cambridge: The MIT Press

[B120] Whittaker J Graphical Models in Applied Multivariate Statistics 1990 Chichester: Wiley

[B121] Li H Gui J Gradient directed regularization for sparse Gaussian concentration graphs, with applications to inference of genetic networks Biostatistics 2006 7 2 302 317 16326758 10.1093/biostatistics/kxj008

[B122] Schäfer J Strimmer K An empirical Bayes approach to inferring large-scale gene association networks Bioinformatics 2005 21 754 764 15479708 10.1093/bioinformatics/bti062

[B123] Wille A Zimmermann P Vranova E Furholz A Laule O Bleuler S Hennig L Prelic A von Rohr P Thiele L Zitzler E Gruissem W Buhlmann P Sparse graphical Gaussian modeling of the isoprenoid gene network in Arabidopsis thaliana Genome Biol 2004 5 11 R92 15535868 10.1186/gb-2004-5-11-r92 PMC545783

[B124] Fan J Feng Y Wu Y Network exploration via the adaptive lasso and SCAD penalties Ann Appl Stat 2009 3 2 521 541 21643444 10.1214/08-AOAS215SUPP PMC3105782

[B125] Friedman J Hastie T Tibshirani R Sparse inverse covariance estimation with the graphical lasso Biostatistics Oxford England 2008 9 3 432 441 10.1093/biostatistics/kxm045 PMC3019769 18079126

[B126] Kiiveri H de Hoog F Fitting very large sparse Gaussian graphical models Comput Stat &amp; Data Anal 2012 56 9 2626 2636

[B127] Witten DM Friedman JH Simon N New insights and faster computations for the graphical Lasso J Comput Graphical Stat 2011 20 4 892 900

[B128] Zou H The adaptive Lasso and its oracle properties J Am Stat Assoc 2006 101 476 1418 1429

[B129] Dudoit S van der Laan M Multiple Testing Procedures with Applications to Genomics 2007 New York: Springer

[B130] Dudoit S van der Laan M Pollard K Multiple testing. part I. single-step procedures for control of general type I error rates Stat App Genet Mol Biol 2004 3 13 10.2202/1544-6115.1040 16646791

[B131] Benjamini Y Hochberg Y Controlling the false discovery rate: a practical and powerful approach to multiple testing J R Stat Soc, Ser B (Methodological) 1995 57 125 133

[B132] Storey J A direct approach to false discovery rates J R Stat Soc, Ser B 2002 64 479 498

[B133] Aubert J Bar-Hen A Daudin J Robin S Determination of the differentially expressed genes in microarray experiments using local FDR BMC Bioinformatics 2004 5 125 15350197 10.1186/1471-2105-5-125 PMC520755

[B134] Efron B Correlation and large-scale simultaneous signifance testing J Am Stat Assoc 2007 102 477 93 103

[B135] Pounds S Morris SW Estimating the occurrence of false positives and false negatives in microarray studies by approximating and partitioning the empirical distribution of p-values Bioinformatics 2003 19 10 1236 1242 12835267 10.1093/bioinformatics/btg148

[B136] Benjamini Y Yekutieli D The control of the false discovery rate in multiple testing under dependency Ann Stat 2001 29 4 1165 1188

[B137] Efron B Size, power and false discovery rates Ann Stat 2007 35 4 1351 1377

[B138] Storey J Tibshirani R Statistical significance for genomewide studies Proc Natl Acad Sci USA 2003 100 16 9440 9445 12883005 10.1073/pnas.1530509100 PMC170937

[B139] Hung JH Yang TH Hu Z Weng Z DeLisi C Gene set enrichment analysis: performance evaluation and usage guidelines Briefings in Bioinformatics 2012 13 3 281 291 21900207 10.1093/bib/bbr049 PMC3357488

[B140] Nam D Kim S Gene-set approach for expression pattern analysis Brief Bioinform 2008 9 3 189 197 18202032 10.1093/bib/bbn001

[B141] Weinberg RA The Biology of Cancer 2007 New York: Garland Science

[B142] Leek JT Storey JD Capturing heterogeneity in gene expression studies by surrogate variable analysis PLoS Genet 2007 3 9 e161 10.1371/journal.pgen.0030161 PMC1994707 17907809

[B143] McClintick JN Edenberg HJ Effects of filtering by Present call on analysis of microarray experiments BMC Bioinformatics 2006 7 49 16448562 10.1186/1471-2105-7-49 PMC1409797

[B144] Bourgon R Gentleman R Huber W Independent filtering increases detection power for high-throughput experiments Proc Natl Acad Sci USA 2010 107 21 9546 9551 20460310 10.1073/pnas.0914005107 PMC2906865

[B145] Carter GW Inferring network interactions within a cell Briefings in Bioinformatics 2005 6 4 380 389 16420736 10.1093/bib/6.4.380

[B146] Perou CM Sorlie T Eisen MB Van De Rijn M Jeffrey SS Rees CA Pollack JR Ross DT Johnsen H Akslen LA Molecular portraits of human breast tumours Nature 2000 406 6797 747 752 10963602 10.1038/35021093

[B147] Gerstung M Eriksson N Lin J Vogelstein B Beerenwinkel N The temporal order of genetic and pathway alterations in Tumorigenesis PLoS ONE 2011 6 11 e27136 22069497 10.1371/journal.pone.0027136 PMC3206070

[B148] Jones S Zhang X Parsons DW Lin JC Leary RJ Angenendt P Mankoo P Carter H Kamiyama H Jimeno A Core signaling pathways in human pancreatic cancers revealed by global genomic analyses Science 2008 321 5897 1801 1806 18772397 10.1126/science.1164368 PMC2848990

[B149] Wood LD Parsons DW Jones S Lin J Sjöblom T Leary RJ Shen D Boca SM Barber T Ptak J The genomic landscapes of human breast and colorectal cancers Science 2007 318 5853 1108 1113 17932254 10.1126/science.1145720

[B150] Vogelstein B Kinzler KW Cancer genes and the pathways they control Nature Med 2004 10 8 789 799 15286780 10.1038/nm1087

[B151] Lazebnik Y What are the hallmarks of cancer? Nature Rev Cancer 2010 10 4 232 233 20355252 10.1038/nrc2827

[B152] Mukherjee S The Emperor of All Maladies: A Biography of Cancer 2011 London: Fourth Estate

